# The histone methyltransferase SETD2 negatively regulates cell size

**DOI:** 10.1242/jcs.259856

**Published:** 2022-10-06

**Authors:** Thom M. Molenaar, Muddassir Malik, Joana Silva, Ning Qing Liu, Judith H. I. Haarhuis, Christina Ambrosi, Eliza Mari Kwesi-Maliepaard, Tibor van Welsem, Tuncay Baubec, William J. Faller, Fred van Leeuwen

**Affiliations:** ^1^ Division of Gene Regulation, Netherlands Cancer Institute, 1066CX Amsterdam, The Netherlands; ^2^ Division of Oncogenomics, Netherlands Cancer Institute, 1066CX Amsterdam, The Netherlands; ^3^ Division of Gene Regulation, Oncode Institute, Netherlands Cancer Institute, 1066CX Amsterdam, The Netherlands; ^4^ Division of Cell Biology, Netherlands Cancer Institute, 1066CX Amsterdam, The Netherlands; ^5^ Department of Molecular Mechanisms of Disease, University of Zurich, 8057 Zurich, Switzerland; ^6^ Life Science Zurich Graduate School, University of Zurich and ETH Zurich, CH-8057 Zurich, Switzerland; ^7^ Genome Biology and Epigenetics, Institute of Biodynamics and Biocomplexity, Department of Biology, Utrecht University, 3584 CH Utrecht, The Netherlands; ^8^ Department of Medical Biology, Amsterdam UMC, University of Amsterdam, 1105AZ Amsterdam, The Netherlands

**Keywords:** SETD2, Histone methyltransferase, Translation, Cell size

## Abstract

Cell size varies between cell types but is tightly regulated by cell intrinsic and extrinsic mechanisms. Cell size control is important for cell function, and changes in cell size are frequently observed in cancer. Here, we uncover a role for SETD2 in regulating cell size. SETD2 is a lysine methyltransferase and a tumor suppressor protein involved in transcription, RNA processing and DNA repair. At the molecular level, SETD2 is best known for associating with RNA polymerase II through its Set2-Rbp1 interacting (SRI) domain and methylating histone H3 on lysine 36 (H3K36) during transcription. Using multiple independent perturbation strategies, we identify SETD2 as a negative regulator of global protein synthesis rates and cell size. We provide evidence that overexpression of the H3K36 demethylase KDM4A or the oncohistone H3.3K36M also increase cell size. In addition, ectopic overexpression of a decoy SRI domain increased cell size, suggesting that the relevant substrate is engaged by SETD2 via its SRI domain. These data add a central role of SETD2 in regulating cellular physiology and warrant further studies on separating the different functions of SETD2 in cancer development.

## Teaser

**Highlighted Article:** Cell size varies between cell types but is tightly regulated. We identify the methyltransferase SETD2 as a negative regulator of global protein synthesis rates and cell size.

## INTRODUCTION

SETD2 is a lysine methyltransferase that is best known for its activity toward lysine 36 on histone H3 (H3K36), which is a histone post-translational modification found on active gene bodies (Li et al., 2016; McDaniel and Strahl, 2017). H3K36 methylation by SETD2 or its homolog Set2 is conserved from yeast to humans and is involved in mRNA co-transcriptional processing, repression of cryptic transcription, and DNA damage repair (Yoh et al., 2008; Luco et al., 2010; Carvalho et al., 2014; Pfister et al., 2014; Mar et al., 2017; Huang et al., 2018; Bhattacharya et al., 2021; Molenaar and van Leeuwen, 2022). In addition, it has recently become clear that SETD2 also methylates non-histone substrates, indicating that SETD2 has functions beyond chromatin regulation (Park et al., 2016; Chen et al., 2017; Seervai et al., 2020; Yuan et al., 2020). SETD2 is frequently mutated in cancer; 4.33% of all cancers carry *SETD2* mutations, with endometrial cancer, renal cancer, bladder cancer and colorectal cancer being most frequently associated with *SETD2* mutations (reviewed by Fahey and Davis, 2017; Lu et al., 2021). Fundamental insights into the functions of SETD2 are required to understand its tumor-suppressor function.

SETD2 is capable of mono-, di- and tri-methylating H3K36 (denoted me1, me2 and me3) *in vitro* through its catalytic SET domain. However, in cells SETD2 is only required for maintaining bulk levels of H3K36me3 but not H3K36me1 or me2 due to the presence of additional H3K36 mono- and di-methyltransferases in mammals (Edmunds et al., 2008; Yuan et al., 2009; Wagner and Carpenter, 2012; Hyun et al., 2017; Li et al., 2019; Zaghi et al., 2020). In contrast, budding yeast only has one H3K36 methyltransferase, Set2, which is responsible for all H3K36 methylation states (Strahl et al., 2002; McDaniel and Strahl, 2017). In addition to its catalytic SET domain, SETD2 contains a conserved Set2-Rbp1 interaction (SRI) domain that binds to the C-terminal domain (CTD) repeats of the largest subunit of RNA polymerase II (RNAPII) when the CTD repeats are phosphorylated at serine-2 and -5 (Sun et al., 2005). This Set2/SETD2–RNAPII interaction is essential for establishing H3K36 methylation on transcribed regions (Kizer et al., 2005; Rebehmed et al., 2014). Based on studies on Set2 in budding yeast, the emerging model is that the interaction between RNAPII and the SRI domain stimulates the activity of the catalytic SET domain rather than that it controls the localization of Set2 to active gene bodies (Youdell et al., 2008; Wang et al., 2015b; Gopalakrishnan et al., 2019). Interestingly, a pathogenic point mutation observed in cancer (R2510H) in the SRI domain of human SETD2 impairs its ability to methylate α-tubulin at lysine 40 during mitosis, whereas global methylation of H3K36 is unaffected by this mutation (Park et al., 2016). Furthermore, it has been recently shown that the SRI domain directly interacts with the acidic C-terminal tail of α-tubulin (Kearns et al., 2021). This indicates that the SRI domain not only controls the activity of SETD2 toward H3K36 but also to non-histone substrates. It also indicates that the role of SETD2 in cancer might involve mechanisms other than defects in chromatin structure.

Methylation of H3K36 has two functions during transcription that are well established in both budding yeast and mammalian cells. First, H3K36me stimulates co-transcriptional mRNA splicing by recruiting splicing factors that ‘read’ H3K36me2 or me3 (Luco et al., 2010; Guo et al., 2014; Sorenson et al., 2016; Leung et al., 2019). Second, H3K36me2 or me3 promotes either the recruitment or activity of chromatin modifiers that repress (cryptic) transcription initiation from within actively transcribed gene bodies (Carrozza et al., 2005; Keogh et al., 2005; Lickwar et al., 2009; Joshi and Struhl, 2005; Baubec et al., 2015; Neri et al., 2017). Another potential function of H3K36 methylation is to promote histone recycling during transcription elongation. Nucleosomes act as barriers for transcription and are therefore transiently disrupted to allow passage of RNAPII (Bondarenko et al., 2006; Petesch and Lis, 2012; Studitsky et al., 2016; Chen et al., 2019). In the wake of transcription, histones can either be recycled or replaced by newly synthesized histones, leading to histone turnover. In budding yeast, Set2 represses histone turnover in active genes, indicating that Set2 promotes histone recycling during transcription (Venkatesh et al., 2012; Smolle et al., 2012; Radman-Livaja et al., 2012). It is currently unclear whether SETD2 has a similar function in mammalian cells. Interestingly, SETD2 promotes both the localization of the conserved histone chaperone FACT (for ‘facilitates chromatin transcription’) to chromatin as well as the maintenance of proper nucleosome organization in active genes in human cells (Carvalho et al., 2013; Simon et al., 2014). Given that FACT promotes histone recycling during transcription in budding yeast (Jamai et al., 2009; Jeronimo et al., 2019) and in *in vitro* studies (Hsieh et al., 2013; Farnung et al., 2021), an attractive model is that SETD2-mediated recruitment of elongation factors such as FACT maintains chromatin integrity (i.e. nucleosome occupancy) during transcription.

Here, we set out to investigate the role of SETD2 in maintaining histone levels. We found that depletion of SETD2 alters the ratio between cellular protein content and histone proteins. This altered histone over total protein ratio was not due to a loss of chromatin integrity leading to global loss of histones from DNA, but rather due to an increase in total cellular protein content and cell size. Protein content is controlled by protein synthesis and degradation rates, and can be coordinated at the level of both transcription as well as translation. Mechanistically, we demonstrate that SETD2 controls global protein synthesis rates, and we provide evidence that this function involves the SRI domain. Thus, although we set out to investigate the role of SETD2 in chromatin integrity, we unexpectedly uncovered a role for SETD2 in cell size control. Cell size is important for cell physiology, and alterations in cell size homeostasis have been associated with disease. How cells tune their size is a fundamental question in cell biology. Each cell type and species has its own characteristic cell size distribution. Cell size across species is maintained cell autonomously and the underlying mechanisms are complex, involving an interplay between tuning biosynthesis and breakdown of nucleic acids, proteins, and other cellular components and programs that control cell cycle, growth and development (Pérez-Ortín et al., 2013; Lloyd, 2013; Amodeo and Skotheim, 2016; Miettinen et al., 2017). Although transcription plays a key role in cell size regulation, how epigenetic mechanisms are involved is still poorly understood. Here, we therefore investigated the role of SETD2 in cell size control.

## RESULTS

### SETD2 controls total protein content and cell size

In metazoans, compromised chromatin integrity leads to the deposition of the replication-independent histone variant H3.3. H3.3 acts as a ‘gap-filler’ histone and prevents the accumulation of naked DNA when histone deposition (e.g. during DNA replication) is compromised (Ray-Gallet et al., 2002; Tagami et al., 2004; Maze et al., 2015; Tvardovskiy et al., 2017). Our initial aim in this study was to determine whether SETD2 represses the deposition of the H3.3 gap filler histone, given that (1) Set2 represses replication-independent histone turnover in active genes in budding yeast (Venkatesh et al., 2012) and (2) SETD2 maintains nucleosome occupancy in active gene bodies (Carvalho et al., 2013; Simon et al., 2014). We therefore depleted SETD2 in human retinal pigment epithelial cells transduced with the telomerase reverse transcriptase gene (RPE1-hTERT). This is a non-transformed near diploid human cell line designated RPE1 from here on. To monitor H3.3 (which differs five amino acids from H3.1 and four amino acids from H3.2), we used RPE1 cells carrying a endogenously V5 epitope-tagged copy of the H3.3 gene *H3F3B* (also known as *H3-3B*) (Molenaar et al., 2020)*.* Despite being frequently inactivated in cancer, SETD2 is an essential gene in several human cell lines (Blomen et al., 2015; Wang et al., 2015a; Bertomeu et al., 2018). Therefore, to prevent looking at potential secondary effects of long-term SETD2 loss, we employed an inducible SETD2-knockdown system using doxycycline (dox) inducible miRNAs against *SETD2* based on the miR-E optimized backbone (Fellmann et al., 2013).

**Fig. 1.**
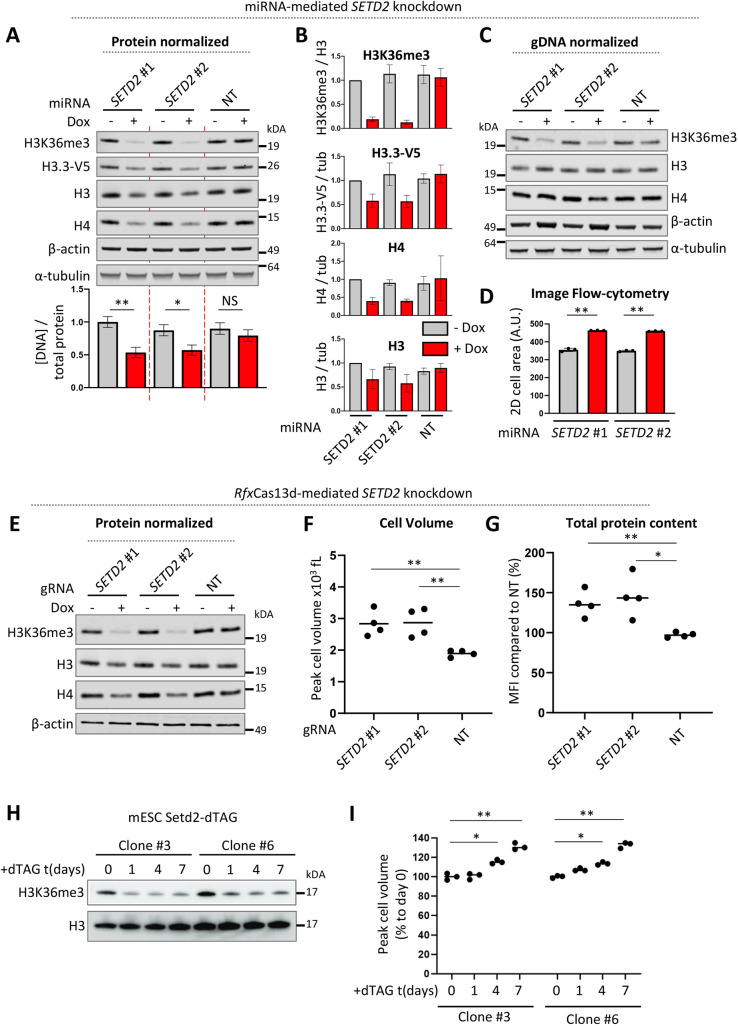
**SETD2 depletion increases cell size and total protein content of RPE1 cells.** (A) Western blot of RPE1 cells with doxycycline (dox)-inducible knockdown of *SETD2* using miRNAs (#1, #2) or a non-targeting (NT) miRNA. Cells were treated with doxycycline for 72 h. The amount of cell lysates added to the gel were normalized for total protein content measured by a Lowry assay. The bar plot below the left panel represents genomic DNA levels quantified by qPCR in protein normalized lysates (mean±s.d.; *n*=3). (B) Quantification of western blot signals in A. Error bars in represent mean±s.d. of three biological replicates. (C) Same lysates as in A, but the amount of cell lysates added to the gel were now normalized for genomic DNA content. (D) 2D cell size (mean±s.d.; *n*=3) as measured by imaging flow cytometry of RPE1 cells with inducible miRNA-mediated SETD2 depletion. Cells were treated with dox for 72 h for inducible miRNA-based *SETD2* knockdown (red). (E) Western blot of RPE1 cells with dox-inducible expression of *Rfx*Cas13d and stable expression of guide RNAs (gRNAs; #1, #2) targeting *SETD2* or a NT gRNA. (F) Cell volume after *Rfx*Cas13d-mediated *SETD2* knockdown (+dox condition). (G) Total protein content after *Rfx*Cas13d-mediated *SETD2* knockdown as measured by flow cytometry of fixed and permeabilized RPE1 cells stained with Zombie NIR amine reactive fluorescent dye. MFI, mean fluorescent intensity. Cells were treated with dox for 96 h in E, F and G. Data in F,G is from four independent biological replicates. (H) Whole-cell lysate western blot of mouse embryonic stem cells (mESCs) with Setd2 endogenously tagged with dTAG. (I) Cell volume of Setd2-dTAG mESCs following SETD2 depletion. Data represents percentage of peak cell volume increase relative to untreated control (day 0); *n*=3. See Fig. S2 for cell volume changes in femtoliter for individual biological replicates. *P<0.05; **P<0.01; NS, not significant (one-way ANOVA with a Tukey's multiple comparisons test). Blots shown in C are representative of three repeats; those in E and H are representative of two repeats. A.U., arbitrary units.

Treating RPE1-*H3F3B*-V5 cells transduced with inducible miRNAs targeting *SETD2* with dox for 72 h led to a reduction in *SETD2* mRNA expression (Fig. S1A) and H3K36me3 levels (Fig. 1A,B), as expected. We first assessed global H3.3–V5 levels in protein-normalized whole-cell lysates from SETD2-depleted RPE1 cells. Unexpectedly, H3.3 levels were significantly reduced when analyzing SETD2-depleted cells (Fig. 1A,B). This was unexpected for two reasons. First, we predicted that SETD2 represses H3.3 deposition in active gene bodies. Second, although a large percentage of the human genome is transcribed by RNAPII (Djebali et al., 2012), only a small percentage (1–5%) of nucleosomes are marked by H3K36me3 in human cells (LeRoy et al., 2013). We therefore did not expect global changes in H3.3 levels upon SETD2 depletion. Strikingly, in addition to H3.3, we also observed that protein-normalized whole-cell lysates from SETD2-depleted cells had reduced histone H3 and H4 levels compared to untreated cells or cells expressing a scrambled miRNA (Fig. 1A,B). Does SETD2 maintain global histone levels (i.e. chromatin integrity) or does SETD2 maintain a normal DNA to total protein ratio? To answer this, we measured genomic DNA levels by quantitative (q)PCR in protein-normalized cell lysates and found that SETD2-depleted lysates had lower DNA levels relative to protein content (Fig. 1A, lower panel). This suggests that the DNA:protein ratio is lowered by SETD2 loss, and that histones appropriately scale with DNA levels in SETD2-knockdown cells. Indeed, when normalizing protein lysates for genomic DNA levels (which equals normalizing for the number of genomes and hence cell numbers), SETD2-depleted cells showed similar histone levels and increased levels of non-histone proteins, such as α-tubulin and β-actin (Fig. 1C). This suggests that SETD2-depleted cells have an increased total cellular protein content.

Global protein levels and cell size are closely correlated. Therefore, the observed protein altered content regulation mediated by SETD2 should presumably lead to an alteration in cell size as well. Indeed, we observed by imaging flow cytometry that miRNA-mediated SETD2 depletion increased cell size (measured as 2D cell surface of cells in suspension) (Fig. 1D). In addition to miRNA-based knockdowns, we suppressed SETD2 expression using an independent alternative approach, *Rfx*Cas13d-mediated RNA cleavage. *Rfx*Cas13d has been reported to have a high knockdown efficiency with minimal off-target effects in human cells (Konermann et al., 2018). Indeed, we observed high mRNA cleavage efficiency using two *SETD2* mRNA targeting guide RNAs (gRNAs; see below) and concomitant loss of H3K36me3 (Fig. 1E). Importantly, *Rfx*Cas13d-mediated knockdown of SETD2 also changed the ratio between β-actin and histones, confirming the miRNA-based SETD2-knockdown results (Fig. 1E). As an independent measurement of cell size, we also directly determined cell volume and observed an increase in cell volume after *Rfx*Cas13d-mediated SETD2 depletion (Fig. 1F). Notably, an increase in cell volume could already be detected 48 h following dox treatment, and cell volume continued to increase with longer treatment time in SETD2 gRNA-expressing cells (Fig. S1B). Finally, to directly and quantitatively measure total cellular protein content, we made use of amine-reactive dyes. When applied to non-permeabilized cells, these dyes stain proteins in the cytoplasm of dead cells and are excluded from live cells. Here, we applied amine-reactive dyes to fixed and permeabilized RPE1 cells to enable staining of intracellular proteins in all cells, while using other flow cytometry parameters to enrich for single live cells (see Materials and Methods). This strategy to directly and quantitatively measure total protein content by flow cytometry confirmed that SETD2 knockdown increased total protein content (Fig. 1G; Fig. S1C). Taken together, these results suggest that SETD2 depletion increases total protein content and cell size/volume in RPE1 cells. We estimate that SETD2-depleted RPE1 cells contain ∼30–40% more protein and have a similar increase in cell volume.

To corroborate our findings, we determined whether SETD2 depletion by independent methods also led to an increase in cell size in an unrelated cell type from a different organism. To this end, we depleted SETD2 in mouse embryonic stem cells (mESCs) by endogenously tagging *Setd2* with the dTAG (degradation tag) (Nabet et al., 2018). In the dTAG system, addition of the small molecule dTAG-13 leads to rapid induction of protein degradation (Nabet et al., 2018). SETD2 was efficiently degraded after treating cells with dTAG-13 as judged by the loss of H3K36me3 (Fig. 1H). Induction of SETD2 degradation led to an increase in cell volume in a time-dependent manner in two independent Setd2-dTAG mESC clones (Fig. 1I; Fig. S2). This indicates that SETD2 loss leads to an increase in cell size in both mouse and human cells in different cell types.

### SETD2 controls protein synthesis rates

**Fig. 2.**
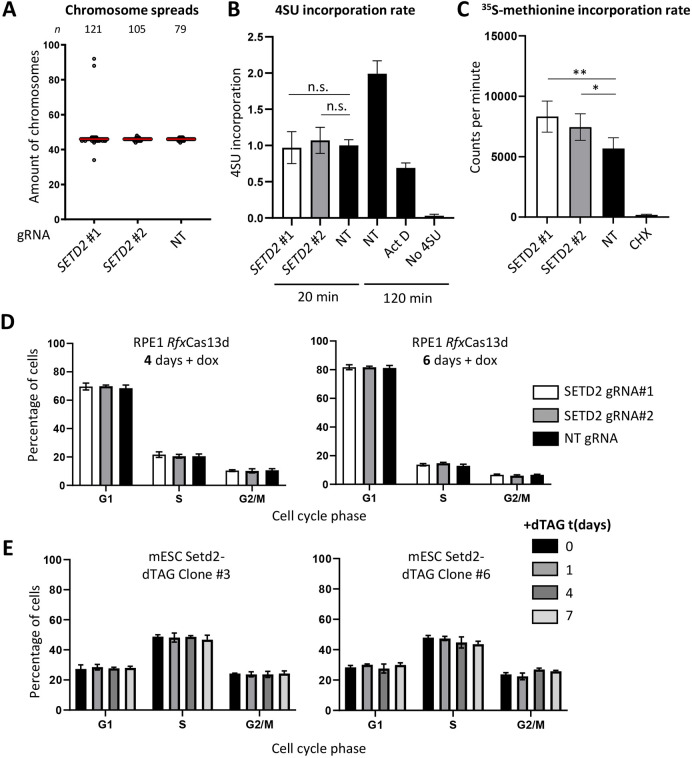
**Inducible depletion of SETD2 increases protein synthesis rates but not ploidy, RNA synthesis rates or cell cycle distribution.** (A) Chromosome counts in pro-metaphase spreads after *Rfx*Cas13d-mediated *SETD2* knockdown or non-targeting (NT) control knockdown. Cells were treated with dox for 96 h. Red line indicates the median. (B) Total RNA synthesis rates as measured by 4SU labeling for 20 min. Equal amounts of RNA were loaded for each sample and 4SU labeled RNA was detected by dot blotting (see Materials and Methods). Longer labeling time increases 4SU signal, whereas treating cells with Actinomycin D (ActD), which is a DNA intercalator that blocks RNA polymerases, reduces the signal. ActD was added at the same time as 4SU and might not act immediately. Cells were treated with dox for 96 h before RNA labeling. (C) [^35^S]Methionine incorporation assay for RPE1 cells 96 h following dox-induced *Rfx*Cas13d-based *SETD2* knockdown. The [^35^S]methionine autoradiography signal was normalized for the total protein content of each condition. CHX indicates a control experiment in which cells were treated with cycloheximide for 1 h, which inhibits protein synthesis. (D,E) Cell cycle distribution as measured by flow cytometry of DAPI-stained SETD2-depleted RPE1 cells (D) and mESCs (E). Cells were treated with dox for 96 h in D. Error bars in B–E are mean±s.d. of three biological replicates. *P<0.05; **P<0.01; n.s., not significant (two-tailed unpaired *t*-test).

Cell volume is normally tightly regulated and can be influenced by perturbations of many different processes. For example, cell volume can be influenced by chromosome ploidy levels (e.g. tetraploid cells are bigger than diploid cells), total mRNA synthesis and translation rates, or a disconnection between cell cycle progression and cell volume regulation (Cadart et al, 2018; Patterson et al, 2021; Amodeo and Skotheim, 2016; Marguerat and Bähler, 2012; Schmoller and Skotheim, 2015). First, we determined whether SETD2 depletion caused an increase in ploidy levels, which is a plausible scenario given that SETD2 controls genome stability through α-tubulin methylation in mitosis. Using the *Rfx*Cas13d system, we found that at 96 h following the start of dox treatment ploidy levels were not significantly altered between SETD2 gRNA and control cells (Fig. 2A). This suggests that SETD2 depletion does not affect cell size by altering chromosome copy number. Secondly, we considered that SETD2 might increase protein content and cell size through regulation of mRNA synthesis. SETD2 is a well-known chromatin regulator and promotes chromatin compaction in the wake of RNAPII transcription. To determine whether SETD2 affects RNA synthesis rates, we monitored nascent RNA synthesis by using the ribonucleoside analog 4-thiouridine (4SU), which is used in transient transcriptome sequencing (TT-seq) to profile nascent transcription (Schwalb et al., 2016). Here, we used 4SU labeling to monitor total nascent RNA synthesis. Dot blotting of 4SU pulse-labeled RNA (normalized for total RNA) revealed that SETD2 depletion did not increase RNA synthesis rates (Fig. 2B). This suggests that SETD2 depletion does not increase global transcription rates, although it could be argued that the majority of newly synthesized RNA comes from RNAPI-transcribed ribosomal RNAs, which could potentially obscure differences in mRNA transcription rates.

Having established that SETD2 likely does not increase cell size through ploidy or RNA synthesis rates, we next turned to protein synthesis rates. An increased cell size can be accompanied by adaptations in protein synthesis and degradation, two opposing but coupled processes. To directly measure protein synthesis rates in SETD2-depleted cells, we used a radioactively labeled [^35^S]methionine incorporation assay. SETD2 depletion using the *Rfx*Cas13d system led to a significant increase in the incorporation rate of [^35^S]methionine normalized for total protein content (Fig. 2C). This indicates that the increased protein content in SETD2-depleted cells is accompanied by an increase in protein synthesis rate.

Cells typically maintain a uniform size when cell cycle progression is altered, but under certain conditions, such as senescence, DNA damage-induced cell cycle inhibition, or palbociclib-mediated CDK4 and CDK6 inhibition, cell cycle progression and cell size control become disconnected (Ginzberg et al., 2018). As such, mammalian cells that are artificially arrested in G1 through CDK4 and CDK6 inhibition and exposed to growth factors generally continue to increase in cell size and have an increased protein synthesis rate compared to proliferating cells (Conlon and Raff, 2003; Ginzberg et al., 2018). We therefore used cell cycle profiling by flow cytometry to determine whether inducible SETD2 depletion led to a G1 arrest. miRNA-mediated SETD2 depletion in RPE1 cells led to a slight increase in the number of cells in G1 and a decrease in the number of cells in S phase and G2 (Fig. S3). *Rfx*Cas13d-mediated SETD2 depletion did not significantly affect the cell cycle distribution of RPE1 cells either 4 or 6 days following dox treatment (Fig. 2D). Similarly, dTAG-mediated SETD2 degradation in mESCs did not alter the cell cycle distribution (Fig. 2E). Collectively, this suggests that SETD2 loss does not consistently alter cell cycle distribution.

**Fig. 3.**
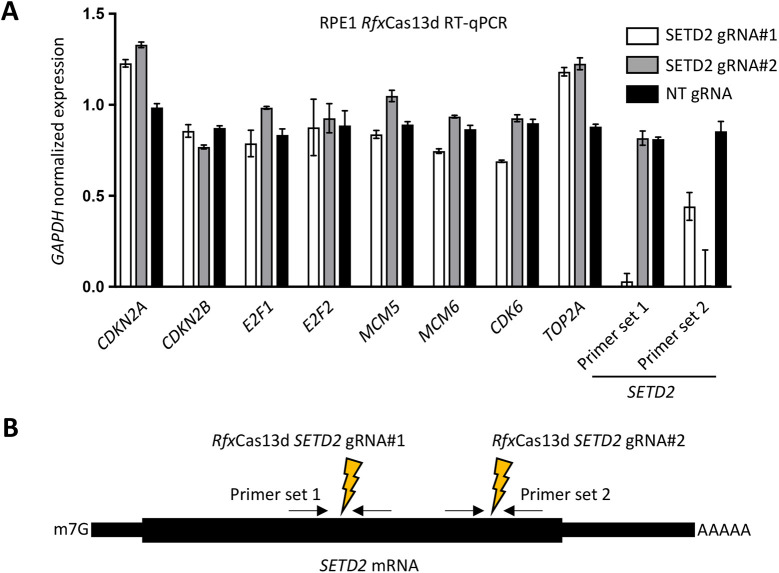
**Expression of cell cycle related genes following SETD2 depletion.** (A) RT-qPCR for mRNA expression analysis of genes involved in cell cycle regulation in RPE1 cells, 96 h following doxycycline-induced *Rfx*Cas13d-based *SETD2* knockdown or non-targeting (NT) control knockdown. (B) The two gRNAs used for targeting *SETD2* mRNA are each flanked by a RT-qPCR primer pair used for *SETD2* expression analysis in A. Error bars represent mean±s.d. of three biological replicates.

To look at potential cell cycle defects in an independent way, we measured the mRNA levels of several genes involved in cell cycle progression in RPE1 cells following *SETD2* knockdown using *Rfx*Cas13d. SETD2 depletion did not substantially affect the expression of genes involved in cell cycle progression such as *E2F1*, *E2F2*, *MCM5*, *MCM6* and *CDK6* (Fig. 3A,B). However, cyclin-dependent kinase inhibitor 2A (*CDKN2A*), which encodes both the p53 stability regulator p14^arf^ and the CDK4/6 inhibitor p16^INK4A^, was upregulated in SETD2 cells. This could be consistent with a G1 arrest induced by CDK4 or CDK6 inhibition and a consequent uncoupling between cell cycle progression and cell cycle regulation similar to what is seen in palbociclib-treated cells. However, since we did not observe an accumulation of cells in G1, it seems unlikely that CDK4 or CDK6 inhibition by p16^INK4A^ is the primary cause of the increase in cell size after SETD2 depletion.

### Mechanistic insights into size control by SETD2

**Fig. 4.**
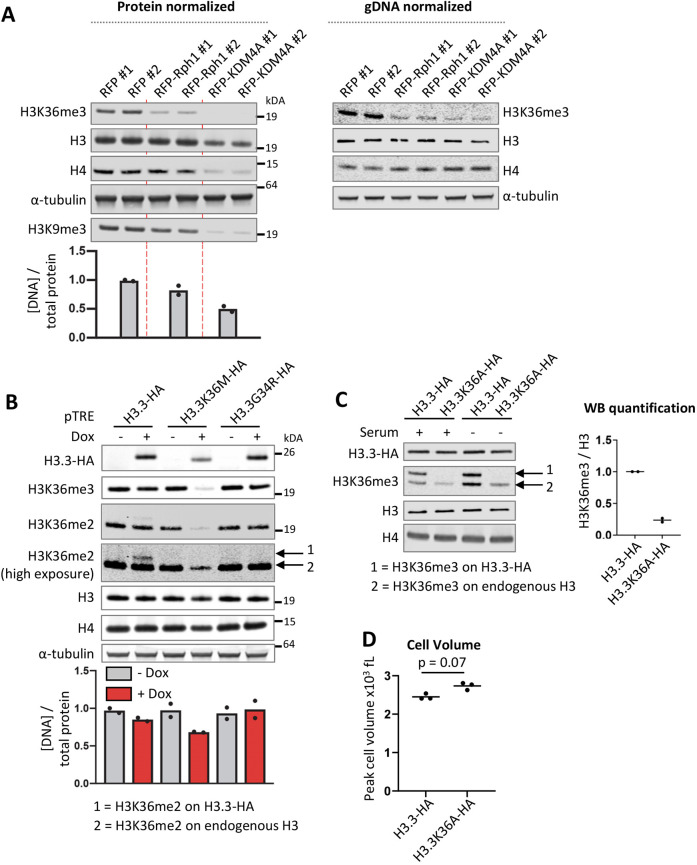
**Mechanistic insights into cell size control by SETD2.** (A) Western blot of RPE1 cells constitutively overexpressing the yeast demethylase Rph1 or human H3K36me3/H3K9me3 demethylase KDM4A. The amount of cell lysates added to the gel were normalized for total protein (left panel) or genomic DNA content (right panel). The bar plot below the left panel represents genomic DNA levels quantified by qPCR in protein-normalized lysates. (B) Western blot of RPE1 cells with doxycycline inducible overexpression of hemagglutinin (HA) epitope-tagged ‘onco’ H3.3 histones. The bar plot represents genomic DNA levels quantified by qPCR in protein normalized lysates. Dots represent the individual values of two biological replicates and blots shown are representative of two repeats (for A,B). (C) Western blot of RPE1 cells with stable overexpression of H3.3–HA and H3.3K36A–HA, with quantification of relative H3K36me3 signal (total signal on H3.3–HA and endogenous H3). Dots represent the individual values of two biological replicates. The arrows 1 and 2 in B and C highlight H3K36me3 on ectopic H3.3–HA and on endogenous H3, respectively. (D) Cell volume of H3.3K36A-overexpressing cells (*n*=3). *P*-value was calculated using one-way ANOVA with a Tukey's multiple comparisons test.

In order to gain mechanistic insights into how SETD2 controls cell size, we first wanted to determine whether SETD2 controls cell size through its catalytic activity. However, we were unable to establish RPE1 cell lines stably (over)expressing an N-terminally truncated version of SETD2 (tSETD2; lacking the first 1400 amino acids, but which is still functional) or a catalytically inactive tSETD2 (with a Q1669A mutation) suggesting that this is lethal in RPE1 cells. As an alternative approach to determine the mechanism through which SETD2 regulates cell size, we stably overexpressed the demethylase KDM4A in RPE1 cells. KDM4A (also known as JMJD2A) counteracts the function of SETD2 on chromatin by converting H3K36me3 into H3K36me2. In addition, KDM4A demethylates the heterochromatin mark H3K9me3. Stable KDM4A overexpression decreased global H3K36me3 and H3K9me3 levels in RPE1 cells, as expected (Fig. 4A). Importantly, KDM4A overexpression increased the total cellular protein content, similar to SETD2 depletion (Fig. 4A). This result suggests that KDM4A might act in the opposite manner to SETD2 to control cell size.

In line with the notion that many chromatin modifiers also act on non-histone proteins, KDM4A has been reported to have functions outside of the nucleus. Specifically, KDM4A associates with the initiating form of the translation machinery and stimulates protein synthesis rates through its catalytic activity (Van Rechem et al., 2015). This suggests that KDM4A-mediated demethylation of a component of the translation machinery might stimulate protein synthesis. However, the identity of this methylated substrate and the methyltransferase involved are unknown. SETD2 is best known for its ability to methylate H3K36, but the list of non-histone substrates that are methylated by SETD2 continues to grow. In an attempt to determine whether SETD2 and KDM4A regulate protein synthesis via H3K36, we also overexpressed the budding yeast homolog of KDM4A, regulator of PHR1 (Rph1) which demethylates both H3K36me2 and H3K36me3 in *Saccharomyces cerevisiae* (Kim and Buratowski, 2007; Klose et al., 2007). Stable Rph1 overexpression in RPE1 cells decreased H3K36me3 levels and had a small effect on H3K9me3 (which is absent in *S. cerevisiae*) but did not significantly alter total cellular protein content (Fig. 4A). One possible explanation for the differential effects between Rph1 and KDM4A overexpression is that they both act on H3K36 but have evolved in conjunction with the opposing methyltransferases (here Set2 and SETD2) to act on additional species-specific substrates. Taken together, these results so far suggest that SETD2 regulates cell size through its methylation activity. However, given the incomplete removal of H3K36me3 in Rph1 overexpressing cells, we cannot definitively determine whether or not H3K36 methylation itself regulates cell size.

To further corroborate our findings, we inhibited SETD2 function by overexpressing the H3.3K36M oncohistone. H3.3K36M, a mutant histone found in chondroblastoma (Behjati et al., 2013), inhibits SETD2 as well as the H3K36 mono- and di-methyltransferase NSD2, in a dominant-negative manner (i.e. *in cis* and *in trans*; Lewis et al., 2013; Lu et al., 2016; Zhang et al., 2017). As a control, we also overexpressed H3.3G34R, which is found in glioblastoma (Schwartzentruber et al., 2012) and osteosarcoma (Behjati et al., 2013), and which inhibits both SETD2 and NSD2 only locally *in cis* (Fang et al., 2018; Shi et al., 2018). Inducible overexpression of HA-tagged H3.3K36M but not H3.3G34R reduced global H3K36me2 and H3K36me3 levels, as expected (Fig. 4B). Interestingly, H3.3K36M overexpression lowered the genomic DNA:protein ratio but not to the same extent as SETD2 depletion or KDM4A overexpression, despite H3K36me3 being almost completely absent. This shows that there is no direct correlation between global H3K36me3 levels and cell size. However, it cannot be excluded that the remaining H3K36me3 localized on a specific set of genes and indirectly regulates protein content.

To more directly investigate the involvement of H3K36 methylation in regulating cell size, we stably overexpressed H3.3 or H3.3K36A in RPE1 cells with the aim to replace a substantial fraction of H3.3 (and canonical H3) with an H3.3K36A histone mutant that cannot be methylated on K36. Humans have 15 genes encoding H3 and H3.3, making it difficult to assess the function of histone modifications by mutating endogenous H3 amino acid residues, a strategy that has been successfully employed in yeast and flies (Meers et al., 2017). Based on H3K36me3 immunoblotting, we found that ectopic expression from the strong *EEF1A1* promoter led to high incorporation levels of ectopic HA-tagged H3.3 (H3.3–HA) (Fig. 4C; Fig. S4A). Note that the C-terminal HA tag interferes with the recognition of the anti-H3 antibody (Abcam, 1791). Since H3.3 accumulates in non-dividing cells (Maze et al., 2015), we also attempted to further increase the level of ectopic H3.3–HA incorporation by depriving RPE1 cells of serum. However, we found that 7 days of serum deprivation did not lead to higher levels of H3.3–HA in RPE1 cells (Fig. 4C). H3.3K36A has been reported to have a minor *trans* inhibitory effect on SETD2 although not as strong as H3.3K36M (Lu et al., 2016). Indeed, we observed that high expression of H3.3K36A–HA (which cannot be methylated and is not recognized by the H3K36me antibodies) reduced H3K36me3 levels on endogenous histone H3 (Fig. 4C). Importantly, H3.3K36A overexpression led to a minor increase in cell volume (Fig. 4D). In contrast to the previous results, this argues in favor of a direct involvement of H3K36 methylation in regulating cell size. The reduction in H3K36me3 levels in H3.3K36A-overexpressing cells was not as strong as that seen in cells with *Rfx*Cas13d-mediated depletion of SETD2 (Fig. S4A), which might explain the minor effect of H3.3K36A overexpression on cell size. It is also noteworthy to mention that the minor *trans* inhibitory effect of H3.3K36A on SETD2 activity might also be responsible for the increase in cell volume. In summary, these experiments confirm that loss of SETD2 activity leads to an increase in cell size, but do not conclusively point toward or against a direct involvement of H3K36 methylation.

### SETD2 controls cell size through its SRI domain

**Fig. 5.**
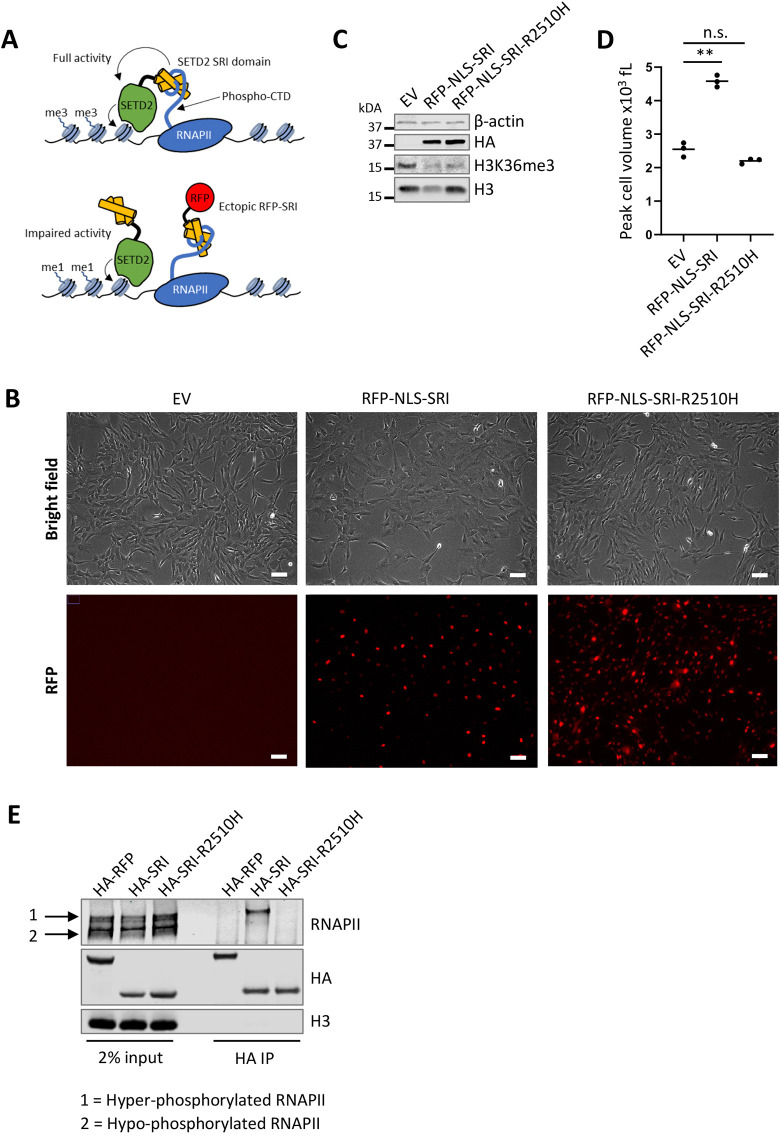
**Ectopic overexpression of the SETD2 SRI domain inhibits H3K36me3 and increases cell size.** (A) Cartoon to illustrate how overexpression of a ‘decoy’ SRI domain might specifically interrupt SETD2 activity toward H3K36 (as well as other SRI-dependent SETD2 substrates). (B–D) Live-cell microscopy (B), whole-cell lysate western blotting (C), and cell volume measurements (D) of RPE1 cells expressing RFP-HA-NLS-hSRI or RFP-HA-NLS-hSRI-R2510H. Cells were transduced with empty vector (EV) or hSRI constructs, selected with puromycin for three days and analyzed six days post-transduction. Scale bars: 55 µm. Images shown in B,C are representative of two repeats. Dots in D are independent biological replicates (*n*=3). **P<0.01; n.s., non-significant (one-way ANOVA with a Tukey's multiple comparisons test). (E) Western blot analysis of ectopically overexpressed SETD2 SRI domains immunoprecipitated from HEK293T cells. HEK293T cells were transiently transfected with HA-NLS-RFP (control), HA-NLS-SRI or HA-NLS-SRI-R2510H encoding plasmids. Cells were lysed 48 h after transfection, and RFP or SRI domains were immunoprecipitated with anti-HA antibody. Blot is representative of two repeats.

As an alternative way to get a mechanistic insight into how SETD2 negatively regulates cell size, we targeted the interaction between SETD2 and RNAPII. This interaction is mediated by the SRI domain, which is conserved from yeast Set2 to human SETD2. The SRI domain interacts with the CTD of RNAPII when phosphorylated at serine 2 and serine 5 in the heptapeptide repeat, and this interaction is essential for establishing H3K36me3 in both yeast and human cells (Kizer et al., 2005; Sun et al., 2005; Rebehmed et al., 2014). Ectopic overexpression of the human SETD2 SRI domain fused to a nuclear localization signal (NLS) reduced global H3K36me3 levels in RPE1 cells, presumably because the excess free SRI domain acts as a decoy for RNAPII (Fig. 5A–C). Overexpression of the yeast Set2 SRI domain had a similar effect (Fig. S4B). Importantly, human SRI overexpression increased cell size (Fig. 5D). This indicates that SETD2 regulates cell size through its SRI domain. To determine whether the nuclear localization of this decoy SRI domain was important for its ability to disrupt cell size regulation, we also overexpressed an SRI domain fused to the HIV Rev protein nuclear export signal (NES). However, we were unable to generate RPE1 cell lines stably overexpressing NES-SRI, suggesting that this is toxic or that NES-SRI is inherently unstable.

Although the SRI domain is best known for its ability to interact with the phosphorylated CTD of RNAPII, the SRI domain also contributes to the ability of SETD2 to methylate non-histone substrates. For example, a pathogenic point mutation in the SRI domain of SETD2 (R2510H) has been reported to disrupt α-tubulin K40 methylation by SETD2 (Park et al., 2016). In line with this, the SETD2 SRI domain was recently shown to directly interact with the C-terminal tail of α-tubulin (Kearns et al., 2021). Although mutating the R2510 residue in the SETD2 SRI domain to an alanine residue (R2510A) disrupts the interaction between the SRI domain and RNAPII (Li et al., 2005), the R2510H mutation disrupts α-tubulin K40 methylation but not H3K36 methylation (Park et al., 2016). Following these observations, SRI-R2510H can be used to functionally separate SETD2-mediated α-tubulin methylation from RNAPII-mediated H3K36 methylation. In contrast to wild-type SRI, SRI-R2510H overexpression did not affect cell volume (Fig. 5D), indicating that cell size regulation by SETD2 is independent of the recruitment of SETD2 by the phosphorylated CTD of RNAPII. However, SRI-R2510H overexpression also affected H3K36me3 levels, although not as strongly as overexpression of the wild-type SRI domain (Fig. 5C). The interaction between SETD2 and RNAPII is required to induce H3K36me3, and SETD2-R2510H can still establish H3K36me3 (Hacker et al., 2015; Park et al., 2016). However, our findings suggest that when ectopically expressed, the R2510H mutation slightly reduces the function of SRI as a decoy for RNAPII. To test whether both versions of the SRI domain bind to RNAPII equally (and therefore act as decoy for SETD2), we immunoprecipitated the ectopic SRI domains from transiently transfected HEK293T cells and found that SRI but not SRI-R2510H interacted with RNAPII (Fig. 5E). This lack of interaction between SRI-R2510H and RNAPII could explain why SRI-R2510H showed a slightly less efficient reduction in H3K36me3 levels upon overexpression in RPE1 cells, as it might not outcompete endogenous SETD2 for RNAPII binding as efficiently as the wild-type SRI domain. In summary, under the conditions used here, SETD2-R2510H did not provide a strict separation-of-function mutation and hence did not provide an unambiguous answer about the involvement of H3K36me3.

## DISCUSSION

SETD2 has multiple cellular functions including RNA processing, the repression of cryptic transcription and DNA repair (Yoh et al., 2008; Luco et al., 2010; Carvalho et al., 2014; Mar et al., 2017; Huang et al., 2018). Mechanistically, most of these processes have been shown to involve the classic molecular function of SETD2, namely, H3K36 methylation. However, as additional non-histone SETD2 substrates continue to be identified, it is becoming clear that the function of SETD2 extends beyond chromatin and transcription regulation (Park et al., 2016; Chen et al., 2017; Seervai et al., 2020; Yuan et al., 2020). Here, we report a novel cellular function of SETD2 in non-transformed human and mouse cells, namely the inhibition of protein synthesis rate and cell size.

An important question that remains is whether the increase in cell size is an indirect consequence of SETD2 depletion, for example via deregulated signaling pathways or cell cycle control, or whether SETD2 controls cell size in a more direct way, perhaps in concert with KDM4A. Although cell size can vary greatly between different cell types, size uniformity is typically maintained in a population of cells of a given type (Ginzberg et al., 2015). Two important mechanisms through which a cell can control its size is the length of the G1 phase of the cell cycle and growth rate (Ginzberg et al., 2018; Liu et al., 2018). Although we did not find differences in cell cycle phase distribution, we cannot exclude that SETD2-depleted cells have altered cell cycle progression dynamics. Previous studies have shown that SETD2 controls S-phase progression through (transcriptional) regulation of dNTP synthesis (Pfister et al., 2015; Pai et al., 2017). One possibility therefore is that a diminished entry into S-phase following SETD2 depletion increases G1 length and thereby increases cell size. However, this does not directly explain the increased cell size that we observed, as cells normally compensate for an increase in G1 length by lowering growth rate (Ginzberg et al., 2018). Alternatively, SETD2 could more directly control cell size through regulation of protein synthesis rate. As mentioned above, KDM4A associates with the translation machinery and stimulates translation through its catalytic activity (Van Rechem et al., 2015). This suggests that KDM4A might stimulate protein synthesis by demethylating a component of the ribosome. Could SETD2 directly negatively regulate protein synthesis by acting opposite to KDM4A in this pathway? Again, it is likely that any direct effect SETD2 might have on protein synthesis rates would be compensated for by a decrease in G1 length to maintain cell size. A more general question might therefore be how SETD2 loss affects the compensation mechanisms that normally maintain cell size. Disruption of CDK4 and cyclin D has been found to decouple cell cycle regulation from size control (Ginzberg et al., 2018). We observed a minor up-regulation of *CDKN2A* expression which encodes both p14^arf^ and the CDK4 and CDK6 inhibitor p16^INK4A^ (Fig. 3A). In addition to careful measurements of G1 length in SETD2-depleted cells, future studies could therefore be aimed at determining whether CDK4 inhibition through p16^INK4A^ or other cell cycle checkpoint regulators contributes to the cell size increase after SETD2 depletion. In this regard, we highlight that SETD2 promotes double-stranded break repair (Carvalho et al., 2014; Kanu et al., 2015; Pfister et al., 2014) and it is therefore conceivable that delayed DNA repair in SETD2-depleted cells leads to prolonged DNA damage checkpoint signaling and inhibition of cell cycle progression.

To determine the mechanism through which SETD2 regulates cell size, it will be important to identify the relevant substrate methylated by SETD2. H3K36me3 is the classical SETD2 substrate and could conceivably regulate the expression of genes involved in translation, for example, by regulating mRNA splicing (Luco et al., 2010; Simon et al., 2014; Leung et al., 2019). Two lines of evidence suggest that SETD2 regulates translation independently of H3K36me3. First, unlike SETD2 depletion, overexpression of the yeast demethylase Rph1 did not affect total cellular protein content. However, because H3K36me3 was not completely abolished in these cells it is possible that local H3K36me3 on certain genes is sufficient to maintain normal protein content. Second, overexpression of the H3.3K36M oncohistone almost completely removed H3K36me3 but did not affect protein content as strongly as *SETD2* knockdown. H3.3K36M acts by inhibiting SETD2 activity *in cis* and *in trans* but it is not completely understood whether H3.3K36M inhibits all SETD2 protein or only the SETD2 protein that has been directed toward H3K36 through its association with RNAPII. In the latter situation, it is conceivable that all activity toward H3K36 can be inhibited by H3.3K36M while there is still SETD2 activity toward substrates other than H3K36 remaining, even though there is less total SETD2 activity available. This could explain why H3.3K36M does not affect protein content as strongly as SETD2 depletion despite a similar decrease in H3K36me3 levels. In contrast, we also obtained evidence supporting the idea that H3K36 methylation might be involved in cell size regulation, as overexpression of H3.3K36A also increased cell volume, although only to a minor extent. This minor effect on cell size could be explained by the fact that H3.3K36A overexpression did not completely remove H3K36me3 from chromatin. This finding still does not provide definitive evidence that SETD2 controls cell size through H3K36me3, as H3.3K36A also modestly inhibits SETD2 *in trans* (Lu et al., 2016), and could therefore conceivably also inhibit SETD2 activity towards a non-histone substrate. We therefore cannot definitively determine at this point whether SETD2 controls cell size through H3K36me3 or through a non-histone substrate.

We provide evidence that SETD2 controls cell size through its SRI domain. This domain not only promotes H3K36me3 levels by controlling SETD2 binding to hyperphosphorylated RNAPII (Kizer et al., 2005) but has recently also been shown to control SETD2 activity to the non-histone substrate α-tubulin (Kearns et al., 2021; Koenning et al., 2021). The increased cell size of cells overexpressing ectopic SRI domain does therefore not directly point towards involvement of H3K36me3. A caveat of this experiment is that SRI domain overexpression might also perturb the binding of other transcription regulatory factors to RNAPII besides SETD2, which could also contribute to disruption of cell size control. An interesting observation we made is that ectopic SRI-R2510H did not bind to RNAPII, which is in contrast to previous reports suggesting that SETD2-R2510H interacts with RNAPII (Park et al., 2016; Koenning et al., 2021). The difference in RNAPII binding of full-length SETD2-R2510H versus SRI-R2510H might therefore indicate that regions outside of the SRI domain also control the interaction between SETD2 and hyperphosphorylated RNAPII.

In support of our findings, a recent study also found a negative role for SETD2 in protein synthesis regulation in clear cell renal cell carcinoma (ccRCC) (Hapke et al., 2020 preprint). SETD2 inactivating mutations are frequently found in multiple types of cancer, including ccRCC (Dalgliesh et al., 2010; Duns et al., 2010; Gerlinger et al., 2012; Sato et al., 2013; Bihr et al., 2019), high-grade gliomas (Fontebasso et al., 2013) and leukemias (Zhang et al., 2012; Zhu et al., 2014; Mar et al., 2014). In addition, SETD2 is mutated at low frequency in many other types of cancers such as melanoma, and lung and colon adenocarcinoma (for reviews, see Li et al., 2016; Fahey and Davis, 2017; Chen et al., 2020). It will therefore be interesting to determine whether an increase in cell size also occurs following mutational inactivation of *SETD2* in cancer cells. Our study warrants further investigation into the molecular mechanism of translation regulation by SETD2, as well as studies to determine whether this function contributes to tumor development in SETD2 mutant or KDM4A-overexpressing cancers. Cell size affects many cellular processes including cell cycle progression, transcription regulation and cell signaling (Marguerat and Bähler, 2012; Neurohr et al., 2019). In addition, as cell size has recently been shown to affect stem cell function (Lengefeld et al., 2021), it will be interesting to determine whether SETD2 affects cellular processes such as differentiation indirectly through its effect on cell size. More generally, our study highlights that alterations in cell size can underlie unexpected phenotypes such as the change in the ratio between cytoplasmic and histone proteins we initially observed in SETD2-depleted cells.

## MATERIALS AND METHODS

### Cell culture, knockdowns and overexpression

Human non-transformed retinal pigment epithelial cells transduced with the human *TERT* gene (*hTERT*-RPE1; ATCC CRL-4000) were grown in DMEM/F12 (Gibco) supplemented with 10% fetal calf serum (FCS). Cells were maintained at 37°C and 5% CO_2_ in a humidified incubator. Mouse embryonic stem cells were cultured in serum-free conditions as described previously (Liu et al., 2021). Cell lines were checked for mycoplasma every 2–3 months.

For microRNA (miRNA)-based knockdown of SETD2, cells were lentivirally transduced with doxycycline (dox)-inducible artificial miRNAs in the miR-E backbone (Fellmann et al., 2013). *SETD2* targeting miRNA sequences were 5′-CCAGGACAGAAAGAAAGTTAGA-3′ (#1) and 5′-ACCGGAAGTTGTTTGAGCAAGA-3′ (#2). Non-targeting miRNA sequence was 5′-CAATGTACTGCGCGTGGAGACT-3′. Knockdown was induced by treating cells with 1 μg/ml dox for 72 h.

For *Rfx*Cas13d-based knockdown of SETD2, RPE1 cells were first transduced with a dox-inducible human codon-optimized *Rfx*Cas13d construct (synthesized by Integrated DNA Technologies; IDT) containing a blasticidin resistance gene. The *Rfx*Cas13d protein sequence, including nuclear localization signal and hemagglutinin (HA) epitope tag, was identical to that described in Konermann et al. (2018). After selection with 10 µg/ml blasticidin (Invivogen), a monoclonal RPE1 cell line showing high *Rfx*Cas13d expression after dox treatment was further transduced with *SETD2* gRNA#1 (5′-AGATCCACAACAAAGACAGCCCA-3′), *SETD2* gRNA#2 (5′-TTCACATTCTCATTGCACTCCAG-3′) or a non-targeting gRNA (5′-TCACCAGAAGCGTACCATACTC-3′) in a construct containing an enhanced green fluorescent protein (EGFP) as a marker. The *SETD2 Rfx*Cas13d gRNAs were designed using the Cas13 guide design resource (Wessels et al., 2020).

For constitutive overexpression of KDM4A (Uniprot ID O75164-1), Rph1 (Uniprot ID P39956), and the SRI domain from *S. cerevisiae SET2*, coding sequences were cloned into a lentiviral vector (pLentiZeo-RFP; Genbank accession number OP471607) in which proteins are N-terminally tagged with tagRFP and expression is driven by the human core *EEF1A1* promoter. Coding sequences were followed by an internal ribosome entry site (IRES) sequence and a bleomycin/zeocin resistance gene. Full-length *KDM4A* was amplified from human RPE1 cDNA. Full-length *RPH1* was amplified from genomic DNA from *Saccharomyces cerevisiae* strain BY4741. The SRI domain from *S. cerevisiae* Set2 (amino acids 619–733) was N-terminally tagged with an SV40 nuclear localization signal (NLS) and codon optimized for expression in humans (synthesized by IDT). For constitutive overexpression of H3.3 and H3.3K36A, codon optimized sequences with C-terminal HA epitope tags (synthesized by IDT) were cloned into the same lentiviral vector but without N-terminal tagRFP. Following transduction, cells were selected with 100 μg/ml zeocin (Invivogen).

For ectopic expression of the human SETD2 SRI domain, DNA encoding SETD2 (Uniprot ID Q9BYW2) amino acid residues 2457–2564 was amplified from RPE1 cDNA and cloned in a lentiviral vector (pLentiZeo-RFP; Genbank accession number OP471607) in frame with N-terminal tagRFP-HA-SV40 NLS and a puromycin resistance gene (replacing the bleomycin/zeocin resistance gene). RPE1 cells were transduced and selected with puromycin (5 μg/ml) for 3 days and analyzed another 3 days later.

For dox inducible overexpression of H3.3, H3.3K36M and H3.3G34R, codon optimized coding sequences with a C-terminal HA epitope tag were synthesized by IDT and cloned into a pCW57.1 (Addgene plasmid #41393) derived lentiviral vector with a blasticidin resistance gene (replacing the original puromycin resistance gene). Following transduction, cells were selected with 10 µg/ml blasticidin (Invivogen) for 7 days. Overexpression was induced by treating cells with 1 μg/ml dox for 96 h.

For generating *Setd2*-dTAG mESC lines, a CRISPR guide targeting near the C-terminal end of SETD2 (Setd2_endo_Cterm_sgRNA1, 5′-AGAGTGACCTCAGGCCAGAG-3′) was used to generate a double strand break (DSB) using CRISPR-Cas9. The DSB was repaired using a donor containing V5-miniAVI-FKBP (Nabet et al., 2018) and flanked by homology arms of 1 kb each. To increase the number of cells with successful homologous recombination, we used a puromycin reporter (Flemr and Bühler, 2015). Genotyping of clones was performed using the following primer pair located on the last Setd2 exon (FWD, 5′-GAGGACCTGGAGTGCAATGA-3′) and on the introduced AVI tag (REV, 5′-GCTCAGAAAATCGAATGGCACGA-3′). Homozygous clones were identified using (REV, 5′-TCAGAGGCAGTAGCCTAGGG-3′) as a reverse primer on the 3′UTR of Setd2, followed by Sanger sequencing of the obtained fragments.

### Cell volume measurements

RPE1 cells were seeded in six-well plates (Techno Plastic Products AG, 92406; 30,000 cells/well) and treated with 1 μg/ml dox for the indicated time points. For mESCs with Setd2-dTAG, 150,000 cells were seeded in 10 cm dishes (Greiner, 664160) and treated with 500 nM dTAG-13 (6605, Tocris). Cells were harvested by trypsinization and resuspended in CASYton (OMNI Life Science, 5651808). Cell volume (fl) was measured as peak of the histogram (PeakVol) on a CASY cell counter Model TT (Innovatis).

### Lentivirus production

Lentiviral transfer plasmids were co-transfected with packaging plasmids pMD2.G (Addgene plasmid #12259), pRSV-Rev (Addgene plasmid #12253), and pMDLg/pRRE (Addgene plasmid #12251) in HEK293T cells (ATCC CRL-3216) using polyethyleminine (PEI, Polysciences 23966) at a 1:3 DNA:PEI ratio. Supernatant was collected 48 h and 72 h post-transfection, passed through a 0.45 µm filter and concentrated using an Amicon Ultra-15 centrifugal filter unit (UFC910024, Merck Millipore).

### RNA isolation and RT-qPCR

For RT-qPCR, RNA was isolated using the RNeasy Mini kit (Qiagen) with on-column DNase I digestion. cDNA was synthesized using Superscript II Reverse Transcriptase (Thermo Fisher Scientific) and random hexamers. For determining *SETD2* knockdown efficiency using the *Rfx*Cas13d system, qPCR primers were designed around the gRNA target site. Primers for qPCR are listed in Table S1.

### Nascent RNA labeling using 4SU

RPE1 cells were seeded in a six-well plate (Techno Plastic Products, 92406; 100,000 cells/well) and treated with 1 μg/ml dox for 4 days. Medium was aspirated and replaced with medium containing 1 mM 4SU (Sigma, T4509). Actinomycin D (1 μg/ml) (Thermo Fisher Scientific, 11805017) was used as a negative control and was added at the moment of 4SU labeling. RNA was harvested using the RNeasy Mini kit (Qiagen) 20 or 120 min after 4SU labeling. Newly synthesized RNA (10 μg) was biotinylated using MTSEA biotin-XX linker (Biotium, BT90066), purified, dot-blotted on Hybond-N membrane (Amersham) and probed with streptavidin-horseradish peroxidase (RPN1231, Cytiva) as described previously (Gregersen et al., 2020).

### Co-immunoprecipitation

Plasmids encoding HA–tagRFP (negative control) or HA–NLS-SRI were transfected into HEK293T cells using Fugene HD at a 1:4 plasmid:FugeneHD ratio in OptiMEM. Cells were harvested in IP lysis buffer (50 mM Tris-HCl pH 7.5, 150 mM NaCl, 5 mM EDTA, 0.5% IGEPAL, 1% Triton X-100) at 48 h after transfection. Lysates were sonicated for 30 cycles at high setting (30 s on, 30 s off) using a Bioruptor Pico sonicator (Diagenode) and centrifuged at 16,873 ***g*** for 10 min at 4°C. Supernatant was used for immunoprecipitation with 5 μg anti-HA antibody (Abcam 18181) overnight at 4°C. Next, immunocomplexes were precipitated with Protein G Dynabeads (Thermo Fisher Scientific, 10004D) for 4 h at 4°C, washed three times with IP lysis buffer, and eluted with SDS loading buffer [50 mM Tris-HCl pH 6.8, 2% SDS, 10% glycerol, 0.1 M dithiothreitol (DTT), 0.02% bromophenol blue]. Samples were boiled, centrifuged (16,873 ***g*** for 5 min) and immunoprecipitated proteins were detected by western blotting.

### Western blotting

For western blotting of RPE1 cells, ∼10^7^ cells were washed twice with phosphate-buffered saline (PBS). Proteins were isolated by adding SDS lysis buffer (50 mM Tris-HCl pH 6.8, 2% SDS and 10% glycerol) supplemented with protease inhibitor cocktail (Roche). DNA was sheared by sonication for 10 min at high settings (30 s on, 30 s off) using a Bioruptor Pico sonicator (Diagenode) to reduce sample viscosity. Protein concentration was determined with the DC protein assay (Bio-Rad) according to the manufacturer’s manual. Samples were supplemented with DTT (final 0.1 M) and bromophenol blue (final 0.02%). Samples were boiled, centrifuged (16,873 ***g*** for 5 min) and 10 μg protein was separated on a NuPAGE 12% Bis-Tris protein gel (Thermo Fisher Scientific) for histones or a NuPAGE 4–12% Bis-Tris protein gel for non-histone proteins. Next, proteins were blotted on 0.2 μm (for histones) and 0.45 μm (for non-histone proteins) nitrocellulose membranes at 1 amp for 90 min. Afterwards membranes were blocked for 30 min with 5% Nutrilon (Nutricia) in PBS and incubated overnight at 4°C with primary antibodies (1:1000 except V5, which was used at 1:5000): H3 (Abcam 1791), H4 (Merck Millipore 04-858), H3K36me3 (Abcam 9050), H3K36me2 (gift from Dirk Schübeler, FMI, Basel; antibody #54R14), H3K9me3 (Abcam 8898), β-actin (Abcam 6276), α-tubulin (Sigma-Aldrich T5168), V5 (Invitrogen R960-25) and HA (Abcam 18181) in 2% Nutrilon in Tris-buffered saline with 0.1% Tween 20 (TBST). The next day, membranes were washed four times with TBST before incubating the membrane with the appropriate Odyssey IRDye secondary antibody (LI-COR Biosciences) at 1:10,000 dilution in 2% Nutrilon in TBST for 1 h. Membranes were washed four times with TBST before scanning on a LI-COR Odyssey IR Imager (LI-COR Biosciences). Signals were quantified using Image Studio software (LI-COR). Western blots involving mESCs (Fig. 1H) were performed as previously described (Liu et al., 2021). Images of uncropped western blots are shown in Fig. S5 (blot transparency).

To normalize protein lysates for genomic DNA concentration, aliquots of protein lysates with equal protein concentration (determined using the DC protein assay, Bio-Rad 5000112) were treated with proteinase K (Sigma, P2308) and RNase A (Sigma, R5000) at 55°C for 30 min, followed by ProtK inactivation at 95°C for 10 min. DNA was ethanol precipitated, washed, dried and resuspended in 50 mM Tris-HCl pH 8. Relative genomic DNA concentrations were determined by qPCR using primers for the *GAPDH* promoter.

### [^35^S]methionine incorporation assay

Protein synthesis rates were measured as described previously (Faller et al., 2015). hTERT-RPE1 cells were incubated with methionine-free DMEM (Thermo Fisher Scientific, #21013024) for 20 min, after which 30 µCi/ml [^35^S]methionine label (Hartmann Analytic) was added for 1 h. After washing the samples with PBS, proteins were extracted with lysis buffer [50 mM Tris-HCl pH 7.5, 150 mM NaCl, 1% Tween-20, 0.5% NP-40, protease inhibitor cocktail (Roche) and phosphatase inhibitor cocktail (Sigma-Aldrich)] and precipitated onto filter paper (Whatmann) with 25% trichloroacetic acid and washed twice with 70% ethanol and twice with acetone. A liquid scintillation counter (Perkin Elmer) was used to measure scintillation and the activity was normalized by total protein content.

### Chromosome counting

RPE1 cells with dox-inducible *Rfx*Cas13d and constitutive expression of gRNAs were treated with 1 μg/ml dox (Sigma, D9891) for 96 h. Cells were treated with nocodazole (250 ng/μl) for 2 h, harvested and subsequently incubated for 10 min in 0.075 M KCl. Samples were fixed with acetic acid with methanol in a 1:3 ratio and DNA was stained with DAPI (1 μg/ml). Samples were dropped on a microscope slide and mounted with a coverslip using Prolong Antifade Gold (Thermo Fisher Scientific). Images were acquired with the Metafer Imaging System and analyzed using Image J (Version 2.1.0/1.53c), images were randomized using the Blind Analysis Tool Plugin.

### Flow cytometry

#### Protein content measurement using amine reactive dyes

Protein content RPE1 clones was measured utilizing the property of amine reactive dyes to stain proteins. Approximately 100,000 cells per condition were stained with 1:1000 Zombie Violet (Biolegend) in PBS to stain dead cells. Cells were subsequently fixed and permeabilized according to the protocol from the Transcription Factor Buffer Set (#562574, BD Biosciences). Intracellular proteins of permeabilized cells were subsequently stained with 1:200 Zombie NIR (Biolegend) in PBS. Cells were centrifuged at 600 ***g*** for 5 min, resuspended in FACS buffer (PBS, 0.5% BSA, 2 mM EDTA) and measured on a LSR Fortessa device (BD Biosciences). Analysis of flow cytometry data was performed in FlowJo (BD Biosciences). Duplets and dead cells were excluded. For protein content the mean fluorescent intensity of Zombie NIR was quantified. Analysis was performed in FlowJo (version 10.5.3).

#### Cell cycle distribution analysis

For cell cycle distribution analysis, hTERT-RPE1 cells were trypsinized, washed with cold PBS, and fixed with 70% ethanol at 4°C for 30 min. Cells were treated with RNase A and stained with propidium iodide (50 µg/ml) (Fig. S3) or alternatively stained with DAPI (1 µg/ml) (Fig. 2D,E). Duplets and dead cells were excluded. Cell cycle analysis was performed using the built-in cell cycle analysis of FlowJo (version 10.5.3), using unconstrained Watson model curve fitting. Alternatively, population-based gating was performed.

### Imaging flow cytometry

For imaging flow cytometry, hTERT-RPE1 cells were detached from culture plates with accutase (Stemcell Technologies) and stained with CellTrace CFSE Cell Proliferation Kit (C34554, ThermoFisher) according to manufacturer's protocol. 2D cell size was measured on an Amnis ImageStreamX Mark II device (Luminex).

### Statistics

Statistical analyses were performed using GraphPad Prism (version 9.1.1). Data are presented as mean±s.d. Sample size was not pre-defined. *P*-values were calculated using the unpaired two-tailed Student's *t*-test or one-way ANOVA with Tukey's multiple comparisons test, as indicated at each figure (*P<0.05; **P<0.01).

## Supplementary Material

10.1242/joces.259856_sup1 Supplementary information
