# Supplementary material for: The histone methyltransferase SETD2 negatively regulates cell size
Source: J Cell Sci. 2022 Oct 6;135(19):jcs259856. doi: 10.1242/jcs.259856 (PMC9659392; doi:10.1242/jcs.259856)
Supplement: Supplementary information [file joces-135-259856-s1.pdf]

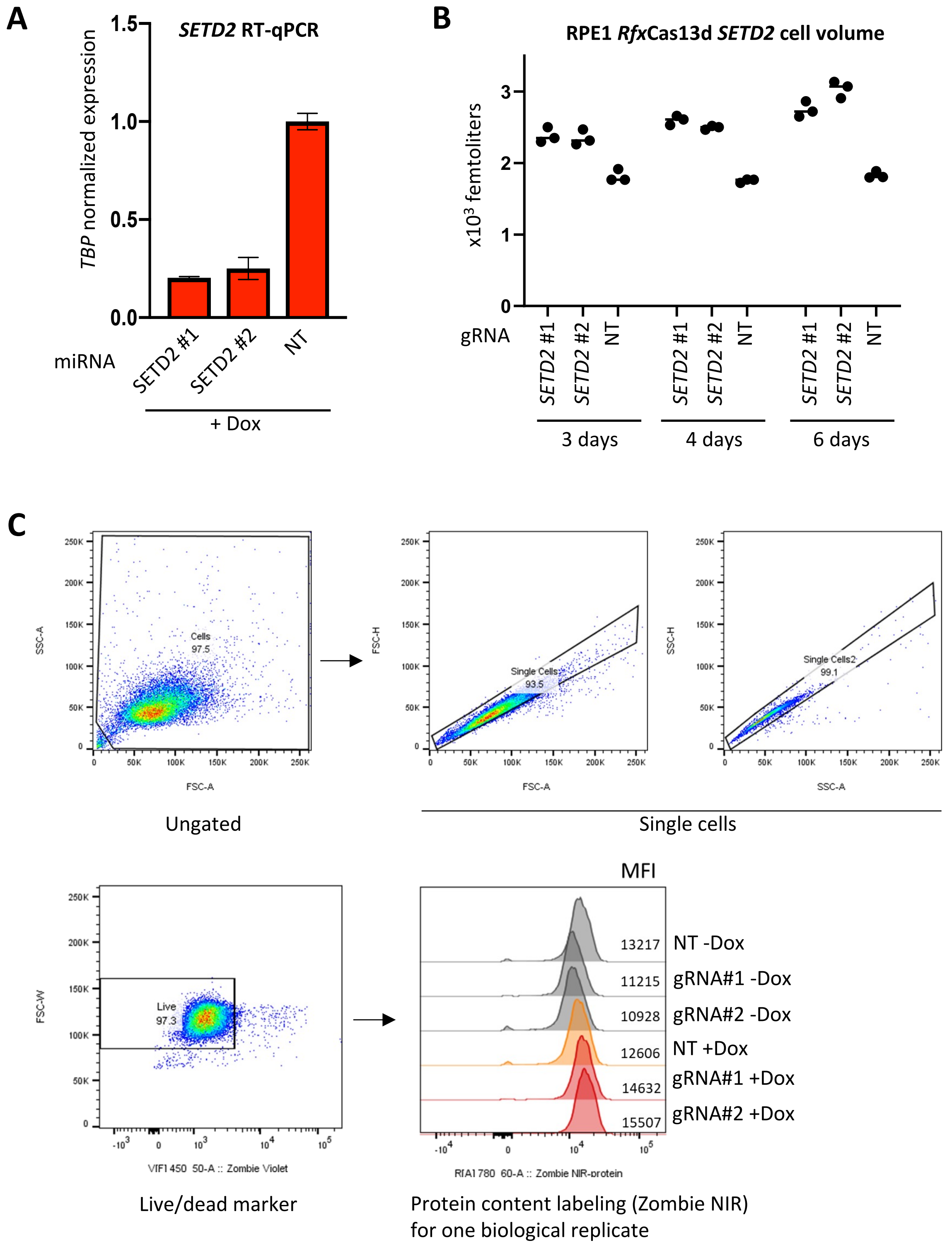

Fig. S1. SETD2 mRNA levels following miRNA-based knockdown in RPE1 cells and cell volume increase after SETD2 depletion in RPE1 and mESC cells. (A) *SETD2* RT-qPCR following miRNA-based depletion in RPE1 cells. Error bars represent mean ± SD of three biological replicates. (**B**) Cell volume after *Rfx*Cas13d-mediated SETD2 depletion in RPE1 cells at different time points. The individual dots are biological replicates. (**C**) Source data for Figure 1G. RPE1 cells were stained with Zombie Violet (to stain dead cells), fixed and permeabilized, and proteins were labeled with Zombie NIR. MFI, mean fluorescent intensity.

Journal of Cell Science • Supplementary information

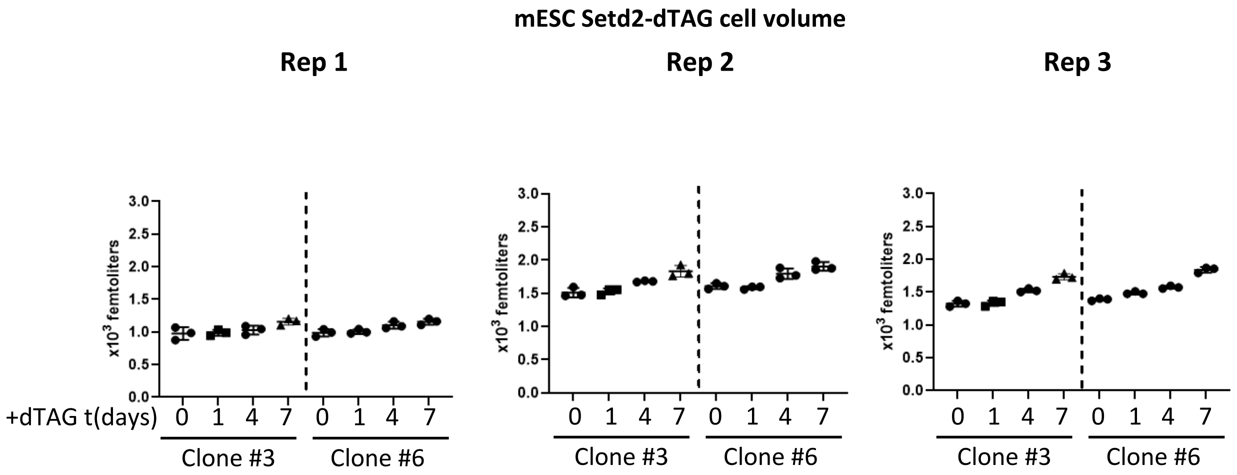

Fig. S2. mESC Setd2-dTAG cell volume changes in femtoliter for three biological replicates. The three plots are the three biological experiments (cells grown and measured on different days). The individual dots are technical replicates (cells grown in different wells of the same plate and measured on the same day).

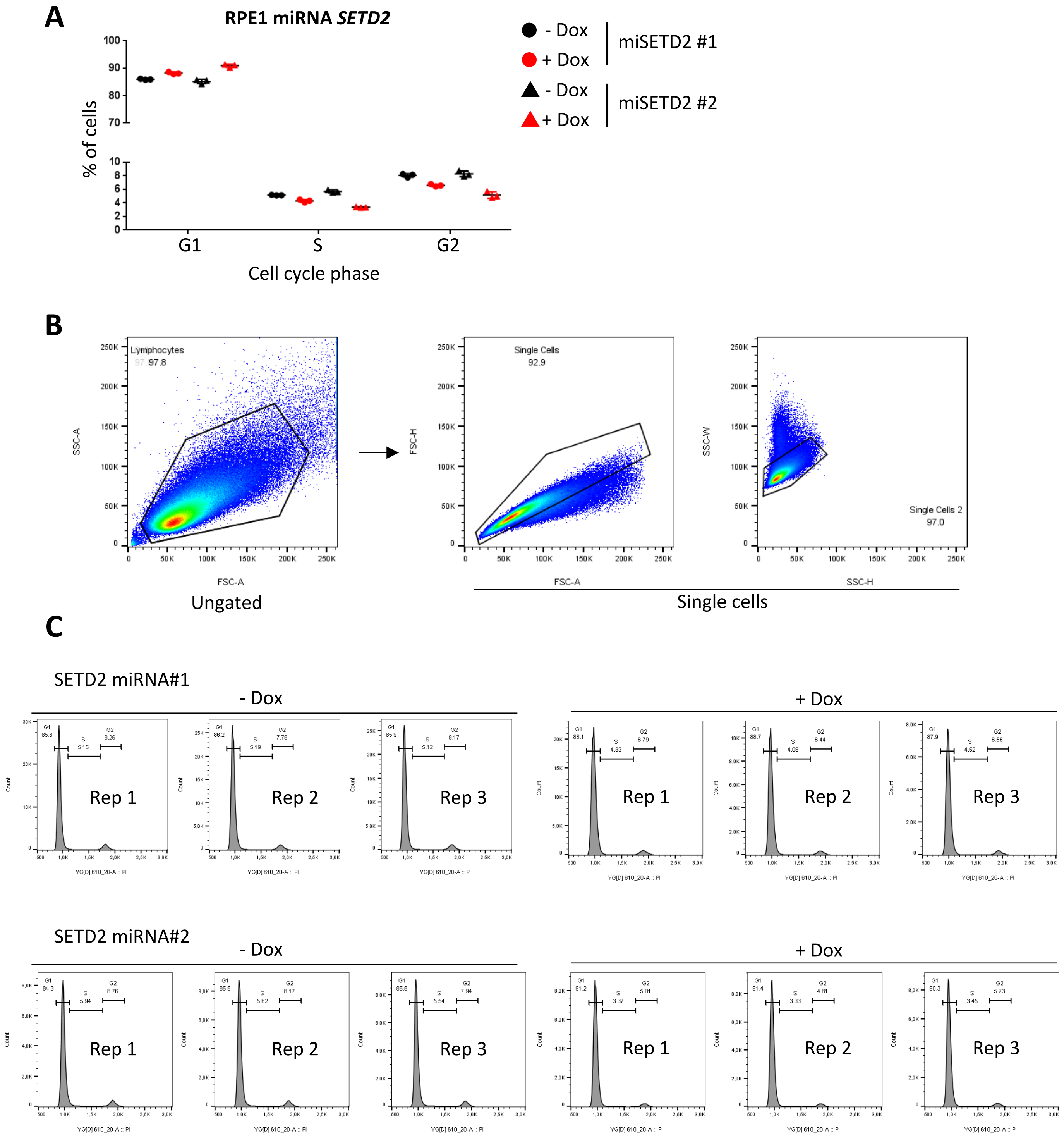

Fig. S3. Cell cycle distribution following miRNA-based SETD2 depletion. (A) Cell cycle distribution after knockdown of SETD2 using dox inducible miRNAs. The individual dots are biological replicates. (**B**) Example of gating strategy for data in (A). (**C**) Source data for (A).

Journal of Cell Science • Supplementary information

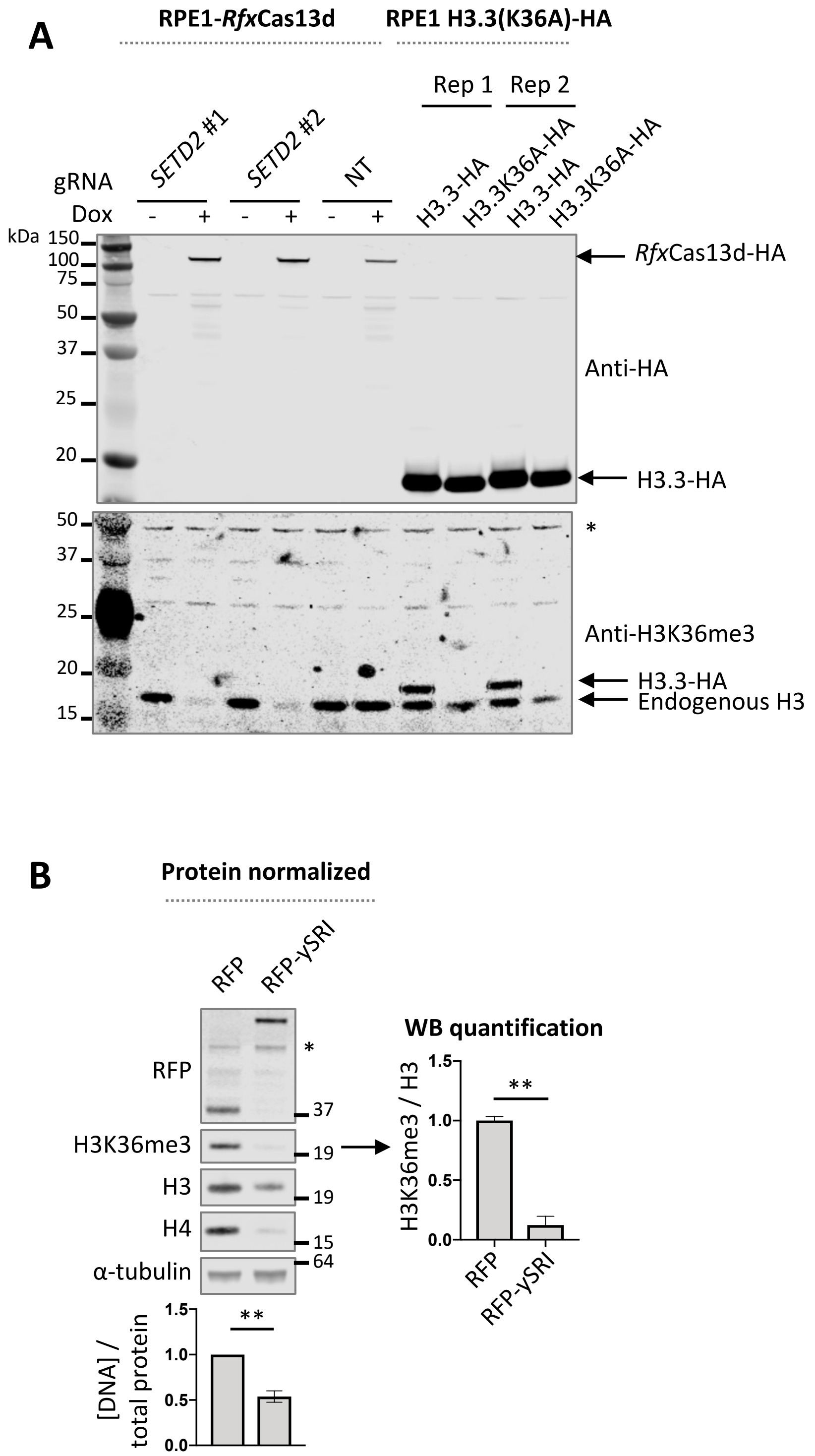

Fig. S4. SETD2 perturbation using H3.3K36A overexpression and overexpression of an ectopic yeast Set2 SRI domain. (A) Western blot to directly compare H3K36me3 levels following *Rfx*Cas13-mediated SETD2 depletion and H3.3K36A overexpression. Rep 1 and Rep 2 indicate the two biological replicates. (**B**) Western blot of RPE1 cells overexpressing the *S. cerevisiae* Set2 SRI domain. Yeast Set2 SRI domain was N-terminally fused to tagRFP and an SV40 nuclear localization signal (NLS). The bar plot below the western blot represents genomic DNA levels quantified by qPCR in protein normalized lysates. Error bars represent mean ± SD of three biological replicates. P-values were calculated using a two-tailed Student's t-test. * $p< 0.05$; ** $p<0.01$.

Fig. S5. Blot transparency. Uncropped western blots of data used for indicated figures.

Source data Figure 1A

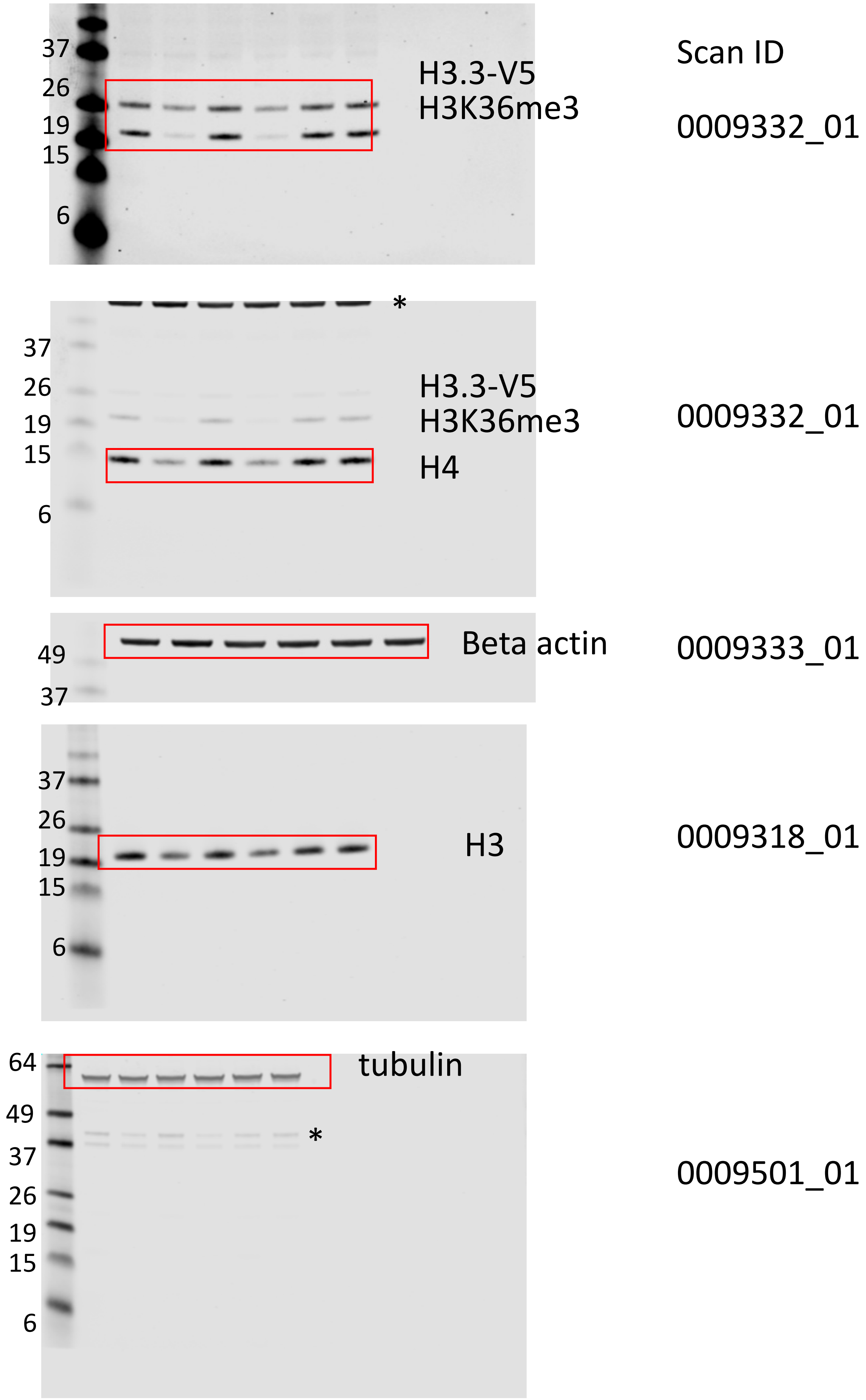

Marker: Benchmark pre-stained protein ladder ThermoFisher 10748010

Journal of Cell Science • Supplementary information

Source data Figure 1C

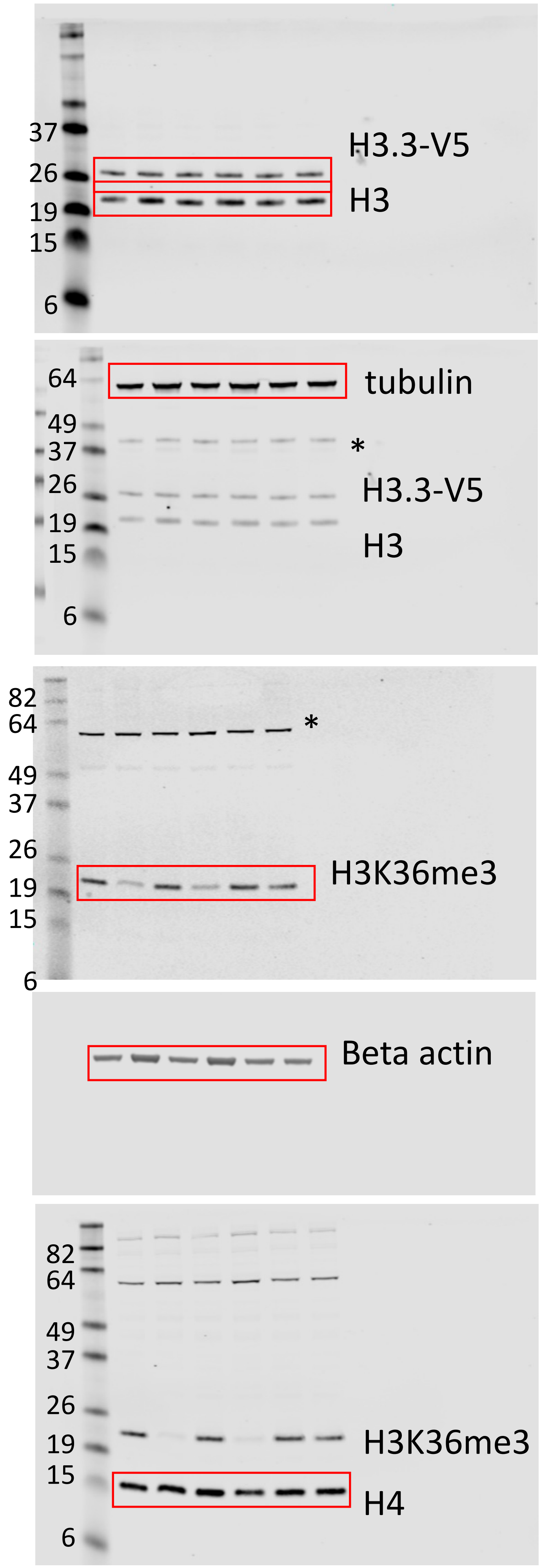

Scan ID

0009895_01

0009912_01

0009999_01

0010035_01

0010022_01

Journal of Cell Science • Supplementary information

Source data Figure 1E

Scan ID

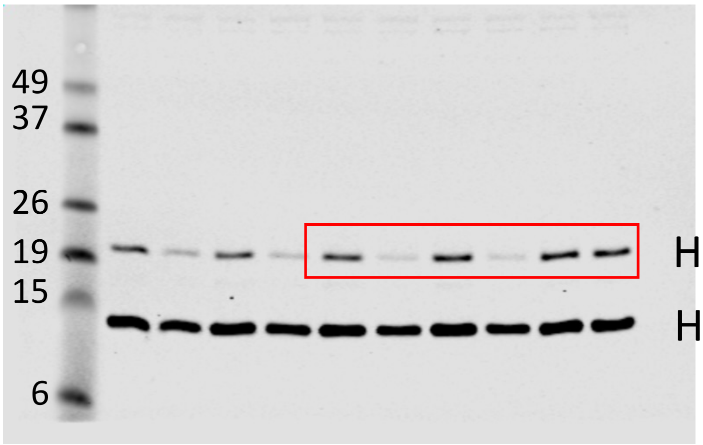

H3K36me3

H4

0009738_01

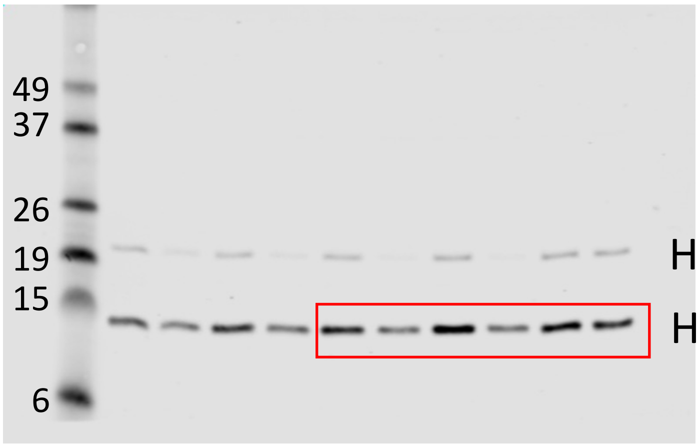

H3K36me3

H4

0009738_01

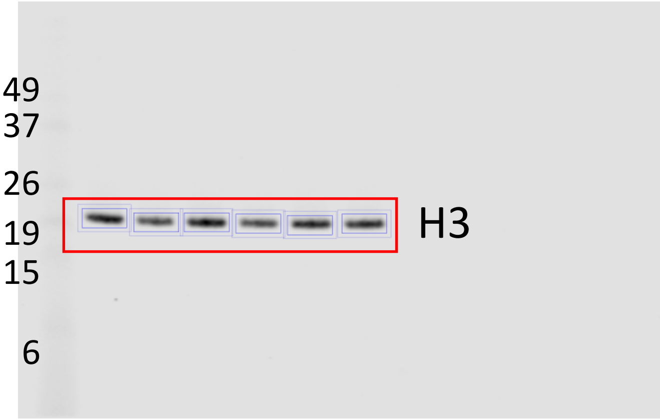

H3

0009776_01

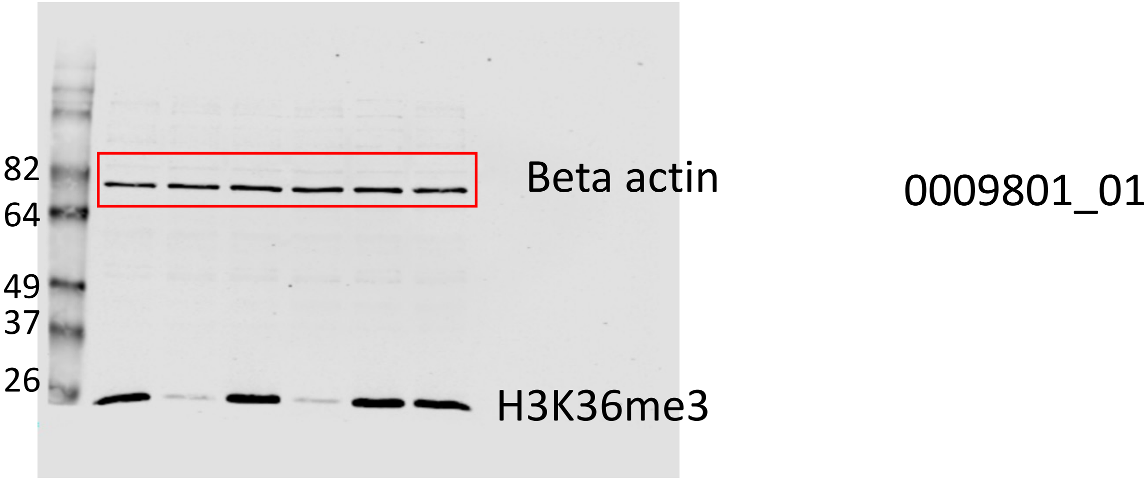

Beta actin

H3K36me3

0009801_01

Journal of Cell Science • Supplementary information

Source data Figure 1H

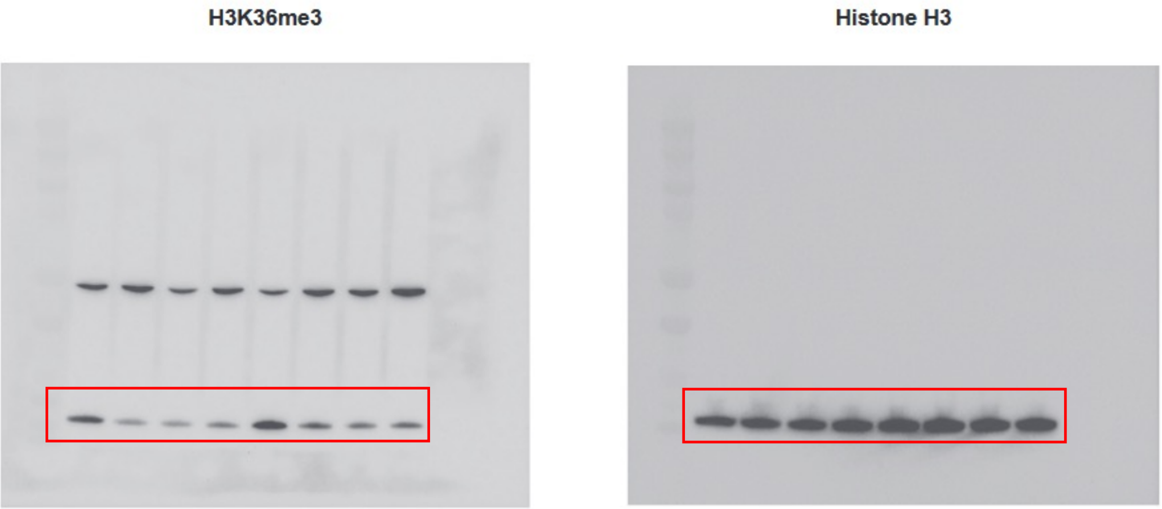

Journal of Cell Science • Supplementary information

Source data Figure 4A

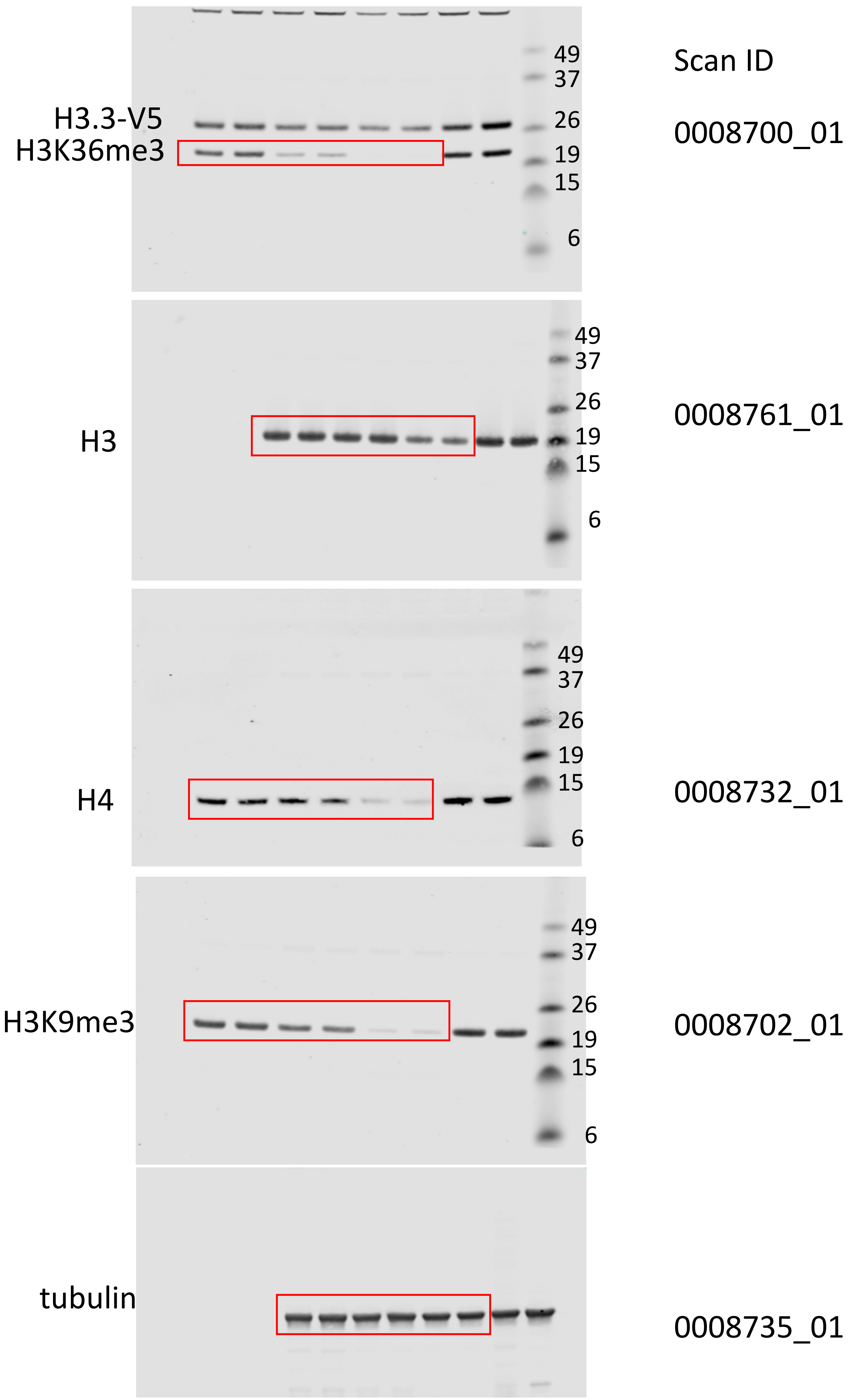

Journal of Cell Science • Supplementary information

Source data Figure 4A

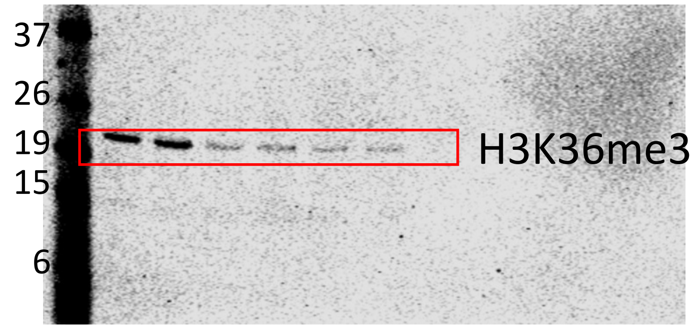

0010002_01

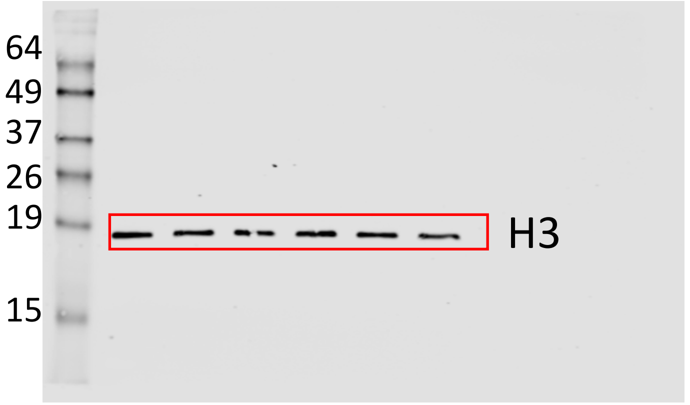

0009994_01

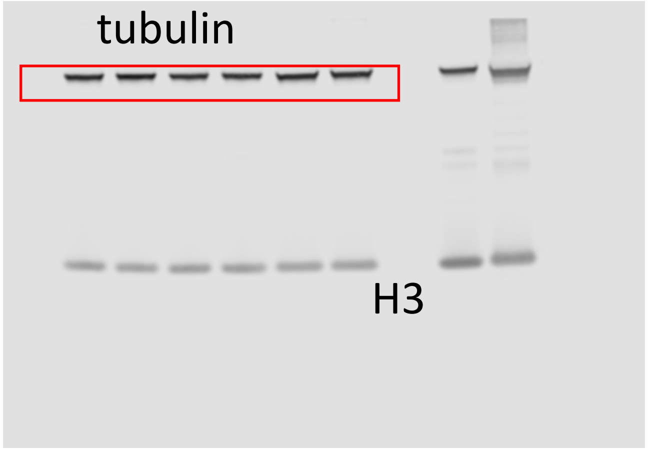

0009735_01

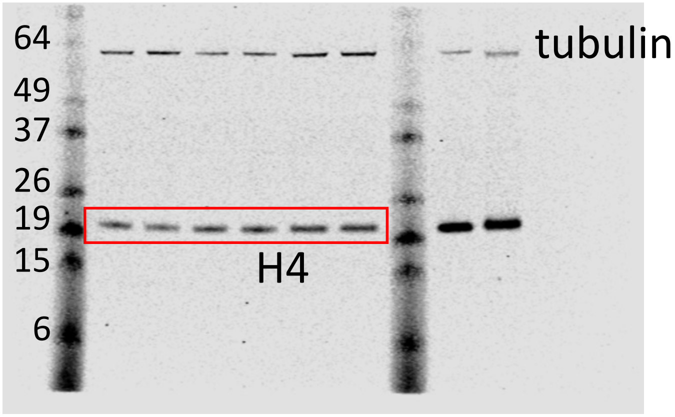

0009733_01

Source data Figure 4B

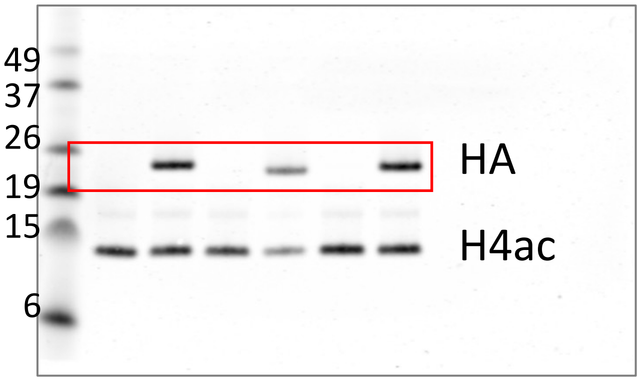

0008582_01

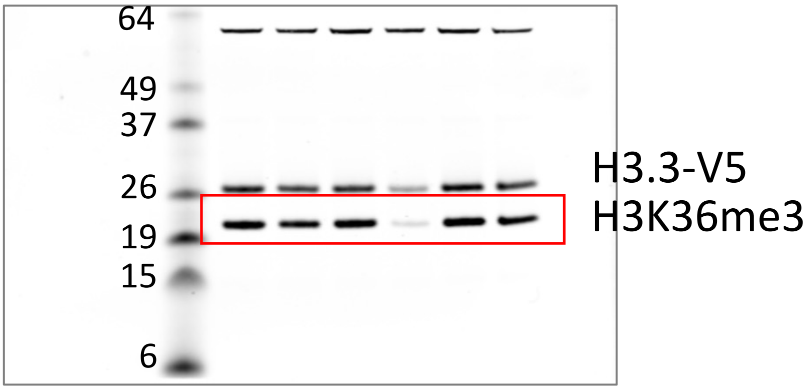

0008581_01

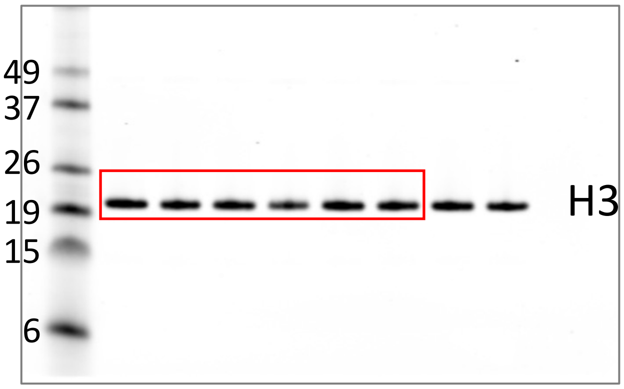

0008591_01

Source data Figure 4B

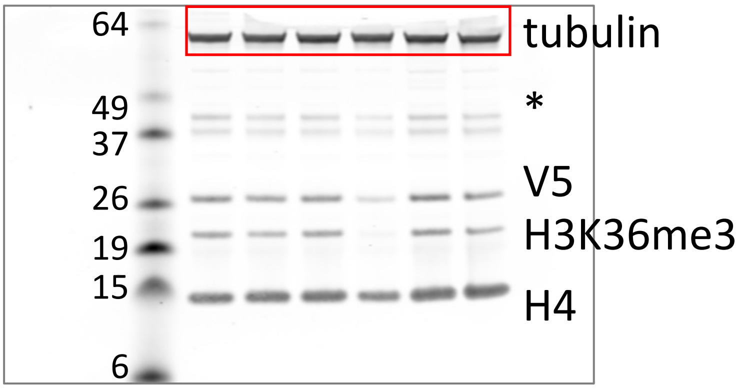

0008593_01

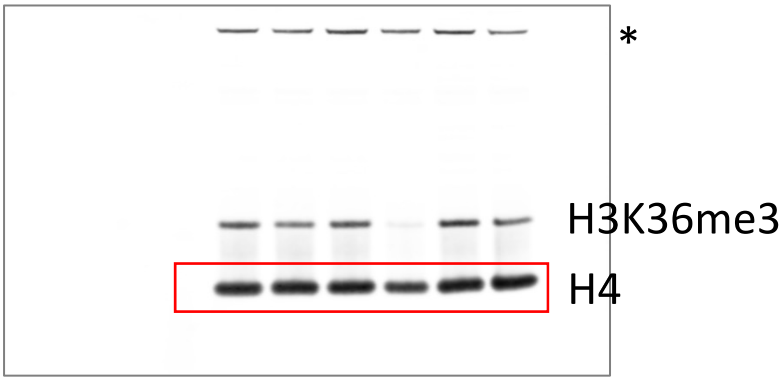

0008585_01

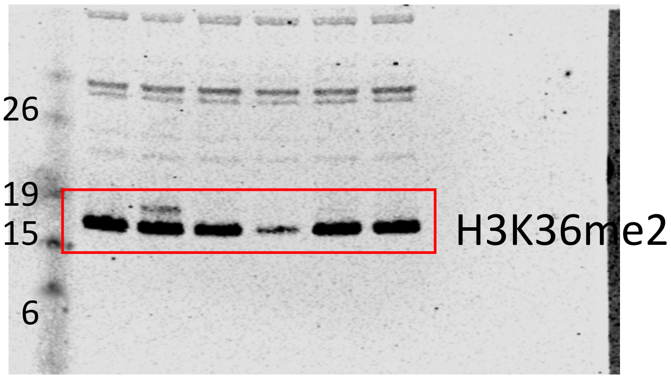

0008665_01

Source data Figure 4C

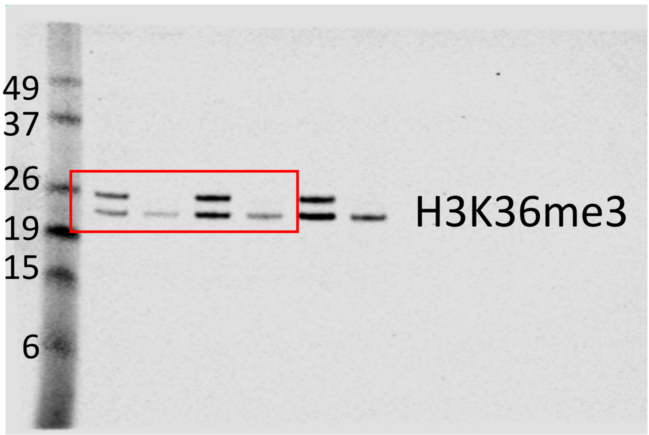

0009588_01

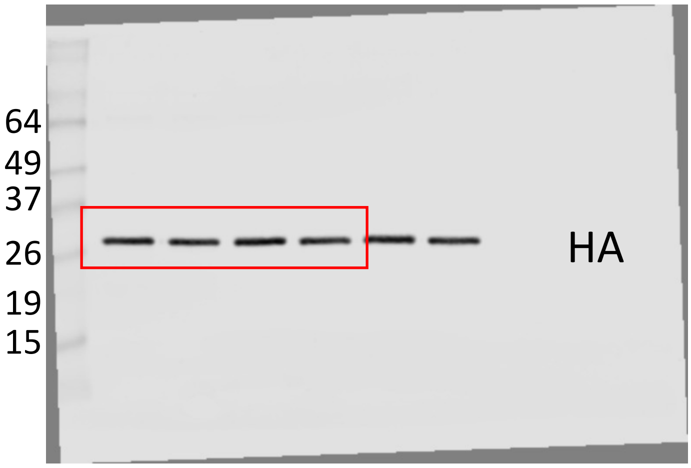

0009619_02

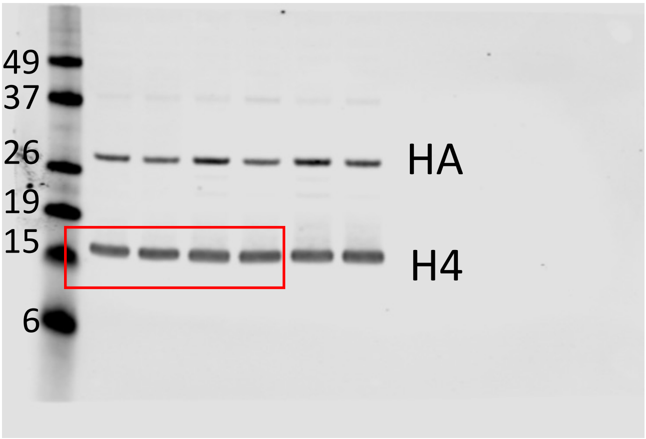

0009601_01

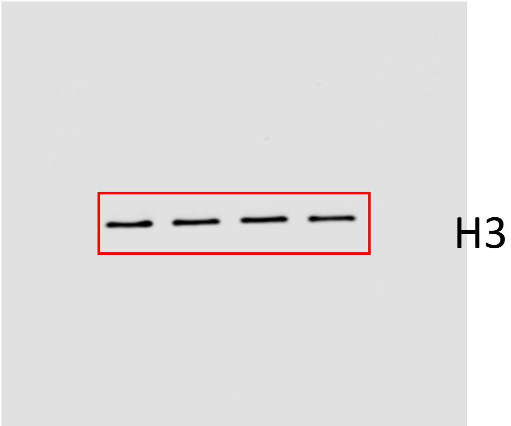

0009976_01

Journal of Cell Science • Supplementary information

J. Cell Sci.: doi:10.1242/jcs.259856: Supplementary information

Source data Figure 5C

Marker: Precision Plus Protein Dual Color Standards, #1610374 | Bio-Rad

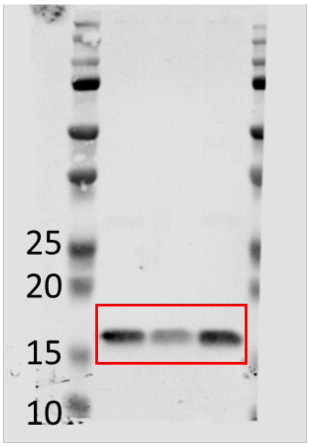

H3

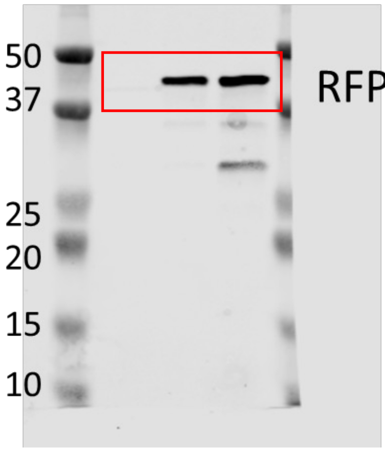

RFP-HA-NLS-SRI (42 kDa)

Anti-HA

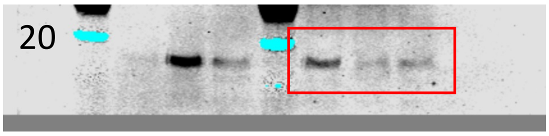

H3K36me3

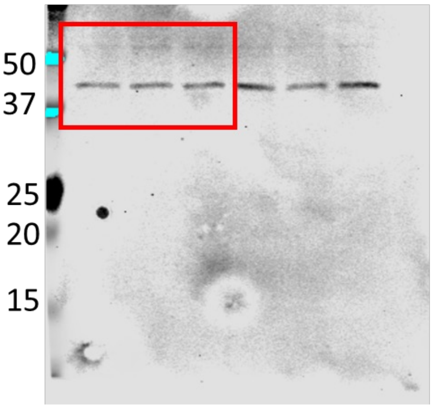

Actin

Journal of Cell Science • Supplementary information

J. Cell Sci.: doi:10.1242/jcs.259856: Supplementary information

Source data Figure 5E

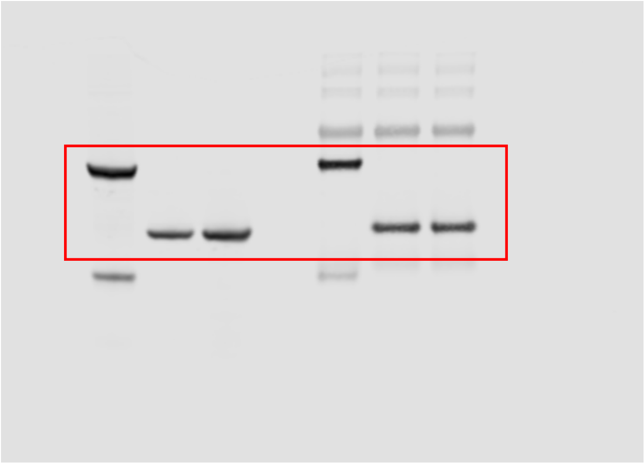

HA
0010053_01

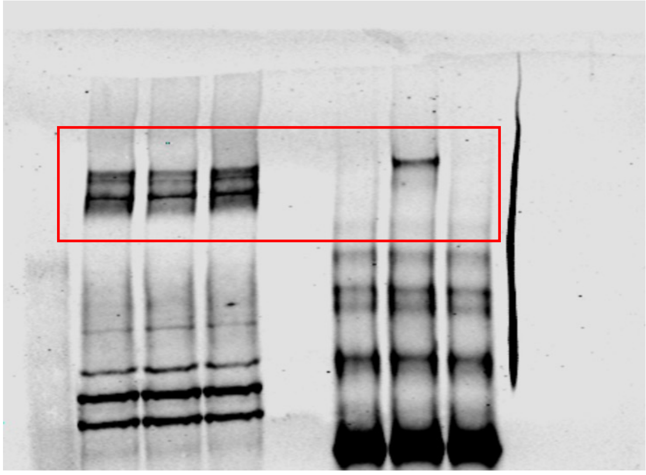

RNAPII
0010044_01

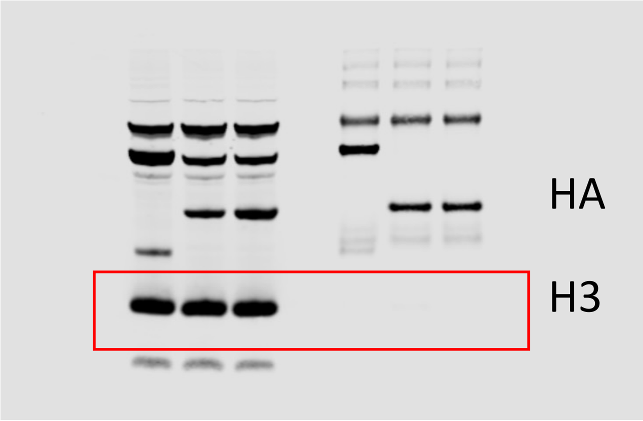

0010082_01

Journal of Cell Science • Supplementary information

Source data Supp Figure 4B

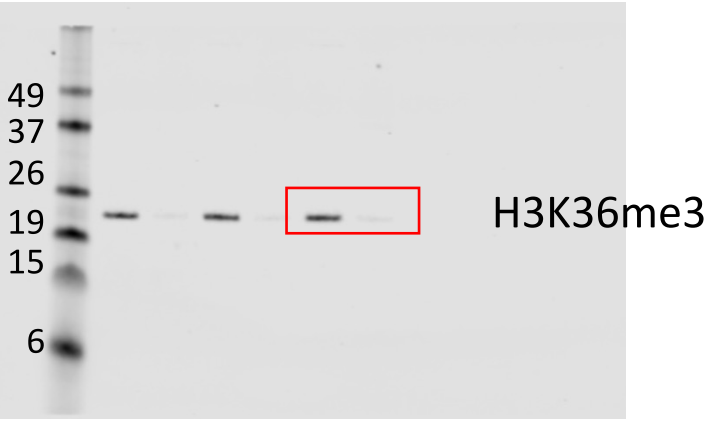

0009621_01

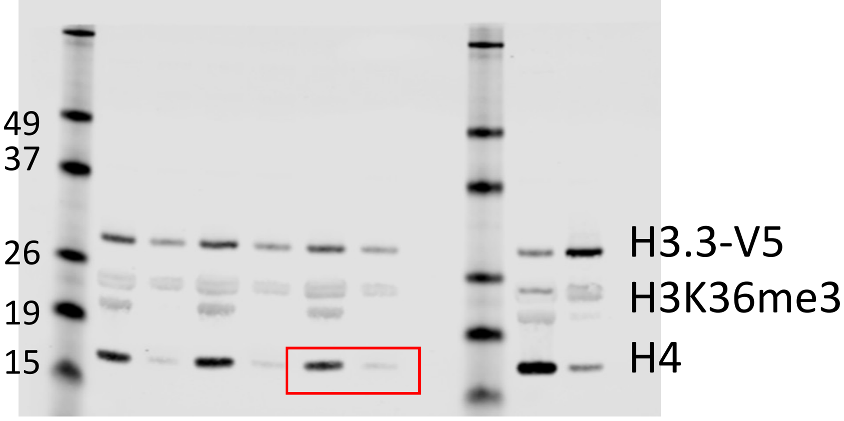

0009522_01

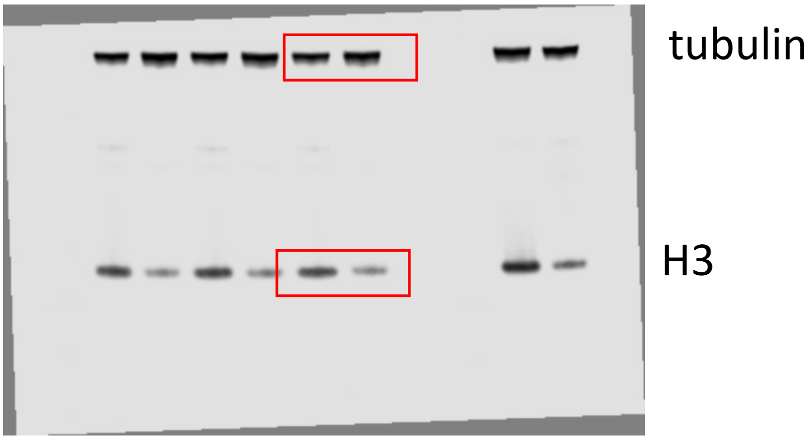

0009523_02

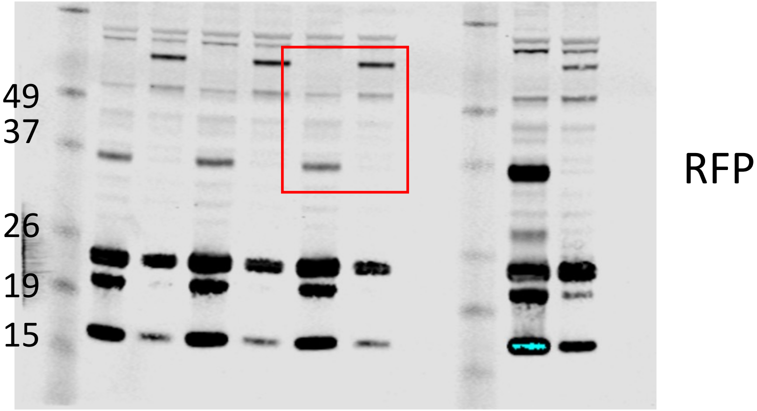

0009548_01

Table S1. Primers used for qPCR in this study.

For RT-qPCR	
CDKN2A_qFwd1	ACTTCAGGGGTGCCACATTC
CDKN2A_qRev1	CGACCCTGTCCCTCAAATCC
CDKN2B_qFwd1	TTTACGGCCAACGGTGGATT
CDKN2B_qRev1	CATCATCATGACCTGGATCGC
MCM5_qFwd1	ATGCAGCGCAAGGTTCTCTA
MCM5_qRev1	GCCAAAAGCACACTTCCCAG
MCM6_qFwd1	GCTCCTGTGAACGGGATCAA
MCM6_qRev1	TACTCAGAGAAGCCCAGCCT
E2F1_qFwd1	CACTTTCGGCCCTTTTGCTC
E2F1_qRev1	GATTCCCCAGGCTCACCAAA
E2F2_qFwd1	CAAGGAAGTCGGTGCAGTCG
E2F2_qRev1	TAGAGATCGCCGCTTGGAGA
CDK6_qFwd1	CCGACTGACACTCGCAGC
CDK6_qRev1	TCCTCGAAGCGAAGTCCTCA
TOP2A_qFwd1	GGCTACATGGTGGCAAGGAT
TOP2A_qRev1	CACGCACATCAAAGTTGGGG
GAPDH_qFwd1	TCAGTGGTGGACCTGACCTG
GAPDH_qRev1	TGCTGTAGCCAAATTCGTTG
SETD2_Rfx_qFwd1	TCAGCTTATCCCGGCTAATGG
SETD2_Rfx_qRev1	TGGGCAAGTGTTCCAAAGTCT
SETD2_Rfx_qFwd2	CCAGTGCCTGAACCCTTACC
SETD2_Rfx_qRev2	GGGTTTGTAAACAGCCCCAA
For DNA quantification	
GAPDH_Promoter_Fw	CTGAGCAGTCCGGTGTCAC
GAPDH_Promoter_Rv	GAGGACTTTGGGAACGACTGA

Journal of Cell Science • Supplementary information
