# Supplementary material for: The histone methyltransferase SETD2 negatively regulates cell size
Source: J Cell Sci. 2022 Oct 6;135(19):jcs259856. doi: 10.1242/jcs.259856 (PMC9659392; doi:10.1242/jcs.259856)
Supplement: Supplementary file 1 [file joces-135-259856_review_history.pdf]

The histone methyltransferase SETD2 negatively regulates cell size

Thom M Molenaar, Muddassir Malik, Joana Silva, Ning Qing Liu, Judith H. I. Haarhuis, Christina Ambrosi, Eliza Mari Kwesi-Maliepaard, Tibor van Welsem, Tuncay Baubec, William Faller and Fred van Leeuwen
DOI: 10.1242/jcs.259856

Editor: Caroline Hill

Review timeline

Submission to Review Commons:	1 November 2021
Submission to Journal of Cell Science:	29 January 2022
Editorial decision:	7 February 2022
First revision received:	19 June 2022
Editorial decision:	8 August 2022
Second revision received:	16 August 2022
Accepted:	23 August 2022

Reviewer 1

Evidence, reproducibility and clarity

The manuscript by Thom M. Molenaar and colleagues reports a novel, unexpected role for the SETD2 methyltransferase in human cells. Through a series of elegant and well- controlled experiments, the authors show that SETD2 negatively regulates protein synthesis and cell size. Although their work did not identify which substrate is involved and how SETD2 inhibits translation, they bring evidence that this function is independent of H3K36 methylation but requires its catalytic activity.

The paper is pleasant to read and well presented. However, I also think that the authors have to bring more compelling evidence to support their conclusions.

Major comments:

1. An important assumption in normalizing to genomic DNA levels to estimate total protein content is that genomic DNA equals cell number. However, this is true only if ploidy stays constant. Although using an inducible system minimizes the risk of ploidy changes, it is important to verify this within the time course of the Dox treatment (3 days) used here.
2. The effects seen upon gDNA normalization are modest (Figure 1 and 3), which can be expected for global changes in cell content / size. It is nevertheless critical to quantify the signal intensities from the Western blots used to build panels C and E, as well as their replicates, in order to give a more accurate view of the size and reproducibility of the effects. The authors should also provide evidence that the Western blotting were done in a semi- quantitative manner, for example by loading one sample at 2-3 dilutions. Also, Figure 3A would be strengthened with an analysis of b-actin levels, which showed stronger effects upon SETD2 knockdown (Figure 1).
3. One argument against a role of H3K36me3 in SETD2 function in protein content regulation is the comparison between the effects seen upon SETD2 knockdown (Figure 1A,D) and overexpression of the dominant-negative H3.3-K36M mutant (Figure 3B). It is difficult to directly compare data from different experiments / Western blots. Indeed, the differential effect on total protein content is weak: ~50% decrease for SETD2 knockdown compared to ~25% decrease for the H3.3 mutant. It is possible that there is the same difference for H3K36me3 levels. It is important to clarify this issue by running the SETD2 KD samples alongside the mutant H3.3K36 samples, on the same gel. Moreover, the experiment using the H3.3-K36A mutant is potentially more convincing, but there are

© 2022. Published by The Company of Biologists under the terms of the Creative Commons Attribution License (https://creativecommons.org/licenses/by/4.0/).

no data showing its effect on total protein content and cell size. Both experiments are critical to substantiate the authors' conclusion that SETD2 controls cell size independently of H3K36me3.
4. Figure 4B shows that overexpression of the yeast Set2 SRI domain increased total protein content, but there are no data on cell size, contrary to the conclusion on lane 318. Although these two readouts are obviously correlated, the paper would be stronger if cell size measurements were provided for key conditions, including the H3.3-K36A mutant and the yeast and human SRI decoy.
5. A key experiment to support a role for SETD2 on protein synthesis would be to verify that SETD2 overexpression has an effect on cell size even if not localized in the nucleus. The authors tried this unsuccessfully with the SRY decoy, but employed a different inducible system (Shield), which may be more leaky than the Dox used in other experiments (Figure 1- 3). A properly inducible SETD2-NES or SRY-NES would bring compelling evidence supporting the central message of this paper. Along this line, is there a properly normalized analysis (eg using spike-in controls) of nascent and total RNA content in ySet2/hSETD2 mutants, which the authors could use as further evidence that SETD2 control translation independently of an effect on chromatin?

Minor comments:

Lane 123: "which differs five amino acids" is missing the preposition "by".

Lane 136: Although observing reduced levels of H3.3 upon SETD2 knockdown is indeed unexpected, I am not surprised by the global effect. Indeed, the human genome is not constituted of "only a small percentage" of "active gene bodies". The ENCODE studies showed that, in human cells for example, a large fraction of the genome is active (> 50%), consistent with the presence of large introns in most genes, which are transcribed and, therefore, decorated with H3K36me3.

It took me a long time to understand that the legends to Figure 1 and 2 are shifted: In the legend to Figure 1, (A) seems to describe what is labelled in panels A-C and (B) appears to describe panels D and E. The data described in (C) appear to be in Figure 2A etc...

I suggest that the authors add a sentence acknowledging that the catalytically active truncated version of SETD2 (tSETD2) only partially restore H3K36me3 levels (Figure 1D) and might therefore not be fully active.

The authors report "a minor decrease in the expression of genes involved in cell cycle progression such as E2F1/2, MCM5/6 and CDK6", which is not clearly visible on Figure 2D. E2F1/2 and MCM5 levels are stable, while MCM6 and CDK6 levels decrease, though upon targeting SETD2 with only one of the sgRNA, not the other. In contrast, the only visible and reproduce difference is a small increase of CDKN2A and TOP2A levels. Please modulate the interpretation of this experiment.

Lane 261: correct "is the they both act".

Can the authors explain why there is no band corresponding to HA-tagged H3K36me2 in the last lane of Figure 3B (upon overexpression of H3.3-G34R-HA)?

The data shown in Figure 4C show that the SRY-R2510H mutation reduces the effect observed with the WT SRY domain, but does not "abolishes" (lane 348) its function. Please rephrase.

Lane 380: for clarity, I suggest to state more explicitly that SETD2 inhibits protein synthesis and cell growth (ie. size).

All statistical analyses were performed using a Student t-test, which is not appropriate to compare more than two means. Rather, an analysis of variance (ANOVA) followed by post- hoc comparisons, which is available in Prism, is designed to compare more than two samples. In addition, the data shown in Figure 3A, 3B and 4C have only two data points and should be reproduced more than once.

All Western blots are missing molecular weight markers.

© 2022. Published by The Company of Biologists under the terms of the Creative Commons Attribution License (https://creativecommons.org/licenses/by/4.0/).

Significance

Overall, this a very interesting manuscript, which brings a new and unexpected contribution to our understanding of SETD2 activities and will be of great interest for the chromatin and cell size control fields. These findings are important because they suggest that SETD2 has functions and substrates beyond its well-characterized roles at chromatin, in methylating H3K36 upon association with elongating RNA polymerase II.

My field of expertise is transcription and chromatin biology.

Referee Cross-commenting

As I am not an expert of cell size regulation and proteostasis, I was not able to to evaluate the experimental approaches used in the manuscript to measure cell size. I think that the concerns brought up by Reviewer #3 are thus very important. Addressing these issues is critical to substantiate the authors' claims and make this paper a significant advance.

Reviewer 2

Evidence, reproducibility and clarity

Summary

This manuscript describes a novel role for the histone methyltransferase SETD2 in regulation of cell size. SETD2 is a conserved methytransferase for histone H3 lysine 36 and is frequently mutated in cancer. It has mostly been studied as the catalyst for methylation of histone H3, although it has other targets in mammalian cells. The authors used conditionally expressed miRNA to knock down SETD2 in RPE1 cells. This led to the expected decrease in H3K36me3 levels, but also decreased total H3 and H4 levels with respect to abundant cytoplasmic protein markers such as actin and tubulin. The authors further showed a decrease in the ratio of DNA to unit protein in SETD2 knockdown cells. Overexpression of a catalytically active SETD2 had the opposite effect. The authors determined that SETD2 knockdown led to increased cell size (measured by flow cytometry) and protein synthesis rates. Overexpression decreased cell size. This effect on cell size was apparently independent of an effect on cell cycle progression. To determine the contribution of H3K36me to these effects, the authors overexpressed demethylase enzymes. A yeast enzyme that specifically acts on H3K36me3 did not affect DNA/protein ratios, whereas KDM4A, which acts on histone and non-histone SETD2 substrates, had a similar effect as SETD2 knockdown. Overexpression of H3.3 K36 mutants failed to phenocopy SETD2 knockdown. In contrast, overexpression of the SRI domain, which directs SETD2 activity to substrates through protein-protein interactions, did affect DNA/protein ratios, presumably by competing with endogenous SETD2. The authors conclude that SETD2 regulates cell size through methylation of a substrate other than H3K36.

Major comments

The manuscript is clear and concise and the data generally support the conclusions. The only "major" comment I have is to provide more clarity surrounding experiments in which SETD2 fragments are overexpressed. Here it is important to know both the levels and subcellular distributions of the overexpressed constructs and how they compare to endogenous SETD2 as there are implications for which SETD2 targets are being affected. For example, the SRI domain overexpression experiments used SRI domain fused to an SV40 NLS-does this mean the decreased DNA/protein ratio is connected to a nuclear target? The Discussion favors other possibilities eg ribosomal proteins. Did other expression constructs also include an NLS? Subcellular localization should be verified for these experiments as well.

Minor comments

-Figure 1 legend is wrong. The description does not match what is in the figure.
-no method provided for determination of protein concentration

© 2022. Published by The Company of Biologists under the terms of the Creative Commons Attribution License (https://creativecommons.org/licenses/by/4.0/).

-Figure 2 legend is out of order
-Figures 3A and B are based on two repeats-are any of these differences significant?
-there is nothing in 3C (or anywhere else) showing the effect of H3.3K36A overexpression on DNA/protein ratio
-more needs to be said about the experiment in Figure 4C. The data as shown are difficult to understand. Why is there no effect of the shield 1 compound on the ability of DD-hSRI expression to phenocopy SETD2 depletion, despite the fact that in the absence of shield 1 expression is barely detected?
-subtle effect of Shield1 compound in 4C for the SRI point mutant is not convincing based on two repeats-need to be able to show the stats

Significance

Identification of a novel SETD2 function that is independent of H3K36me is a significant conceptual advance. The finding could have broad implications for understanding non- canonical functions of histone modifying enzymes and should be interest to those studying these important regulators.

My expertise: histone modifications, chromatin, transcription

Reviewer 3

Evidence, reproducibility and clarity

Summary:

In this manuscript, Molenaar, et al. investigate the role for SETD2 (a lysine methyltransferase) in cell size regulation. They show that SETD2 depletion changes the ratio of genomic DNA to total protein and ascribe this to changes in cell size. They then set out to uncover whether the catalytic activity of SETD2 and the SRI interaction domain are required for mediating this phenotype. The authors proceed to conclude that the role of SETD2 in regulating cell size requires its catalytic activity but is independent of H3K36 methylation.

While the separation of function experiments that were conducted to probe the importance of the SRI domain were clever and well done, there are several issues with the way the effect of SETD2 on cell size is quantified, making it unclear how much of a role SETD2 truly has on regulating cell size. Moreover, the authors do not directly show that SETD2's catalytic activity is required for this observation, making it difficult to draw the conclusion that SETD2 regulates cell size through its catalytic activity. Together, these issues bring into question the magnitude of the effect of SETD2 on cell size and the mechanistic basis for how this mode of regulation occurs.

Major Comments:

1) Size measurements should be made directly and not by comparing protein ratios. The effect of SETD2 on cell size is an extremely important point in this manuscript, however there is only one figure (Figure 2A) that shows any measurement of this. Moreover, the technique used for this (imaging flow cytometry) is not explained either in the text or in the methods and deviates from those that are commonly found in publications about cell size (e.g., Coulter Counter-based measurements, QPI, or measurement of protein content using amine-reactive dyes). Because cell volume is relevant here, it would be important to include measurements of cell volume (instead of just area). Based on the provided measurements, it is not intuitive what the indicated difference in size means relative to the control cells (e.g., how many times bigger are the SETD2 knockdown cells relative to the control?). It would be important for the authors to show this phenotype using another method and to include these measurements in all experiments that claim changes in cell size.
2) Total protein quantifications: The authors show that the effect on cell size correlates with an increase in protein content and translation rates. However, cell size and protein abundance are not synonymous measurements, and one should not be used as a proxy for the other (as is the case in Figure 4B or lines 273-276, for example). Moreover, the measurement of total cellular protein by normalizing to genomic DNA could be more convincingly made by Coomassie staining rather than by looking at the loss of histone proteins when gel loading is normalized to protein content vs.

© 2022. Published by The Company of Biologists under the terms of the Creative Commons Attribution License (https://creativecommons.org/licenses/by/4.0/).

maintenance of histones when normalized to genomic DNA. As mentioned in the previous comment, amine-reactive dyes may also be useful for this.
3) The authors indicate that SETD2 controls cell size through its catalytic activity, however this is not shown directly. They instead show opposing effects when KDM4A (a demethylase with activity that counteracts SETD2) is expressed. While these results are promising for their proposed mechanism, they do not directly show that SETD2's catalytic activity is the driver of the observed phenotype. Moreover, the effect of KDM4A on protein synthesis is already established (Van Rechem et al. 2015), therefore this observation does not add any new insight. As such, it is misleading to definitively assign importance to SETD2's catalytic domain based on these data. While the authors indicate they had trouble generating a stably overexpressing catalytically inactive tSETD2, they may consider generating a doxycycline- inducible one instead.
4) The authors focus on total cellular protein abundance changes when SETD2 is knocked down but do not address the possibility that SETD2 is globally changing transcript abundance, which is in turn translating to a difference in protein amount. This seems especially important given SETD2's known relationship with RNAPII. Mechanistically, it would be important to show that SETD2 does not affect global transcript levels before focusing on total protein content.
5) The authors show that bulk translation is increased in SETD2 knockdown cells. It would be important to establish whether this reflects an overall increase in protein synthesis due to increased size (with no change in the translation rate of specific transcripts) or if this increase is a specific effect that affects particular proteins.

Minor Comments:

1) The figure legends are off in a few places in this manuscript (e.g., Figure 1B and C do not match the legend, and there is no legend for Figure 1D-E; Figure 2 starts at 2B but is labeled as 2A)

2) In the text, the authors indicate that there is a small decrease in the expression of E2F1/2, MCM5/6, and CDK6 when SETD2 is knocked down, but this is not what the bar plot in Figure 2D shows. Moreover, there seems to be a fairly robust increase in CDKN2A and TOP2A when SETD2 is knocked down, but this is not addressed in the text. Particularly because CDKN2A encodes CDK inhibitors that cause G1 cell cycle arrest, this seems like an important consideration given that the authors are trying to show a negligible effect on G1 duration. Based on these discrepancies, the cell cycle analysis that was conducted in Figure 2 seems to be inconclusive.

3) The method for how the cell cycle profiles were obtained in Figure 2C is not described in the text or in the methods. The raw FACS data for this (as well as the imaging flow cytometry experiment) should be included in the supplement.

4) The initial description of SETD2's effect on the DNA: protein ratio is a bit confusing (lines 141-149). By stating that they ..."found that SETD2 depleted lysates had lower DNA levels", it seems to imply that SETD2 KD cells are losing DNA (which is not the case). The description of these experiments could be clarified.

5) The decrease in histones H3 and H4 described in the text and shown in Supplemental Figure 1C is not clearly visible by eye; thus, authors should include quantifications for multiple replicates if they wish to make the claim that this decrease is observable in multiple cell lines.

6) There are several bar plots (e.g., Figure 1B, 2B, 2C) where the error bars are not identified (e.g., SEM or SD) and it is not indicated how many replicates were performed for these experiments.

7) In line 293, the authors state that "H3.3K36A did not affect cell size," however I cannot find data in the figures that support this claim.

8) The serum starvation experiment described in lines 287-290 is informative but as it is a negative result, putting it in the supplement may help streamline the text a bit.

9) For western blots that are normalized by genomic DNA, the authors should include the GAPDH qPCR results in the supplement.

© 2022. Published by The Company of Biologists under the terms of the Creative Commons Attribution License (https://creativecommons.org/licenses/by/4.0/).

Significance

Nature and significance of the advance:

There are many inconclusive aspects of this manuscript that make it difficult to determine the magnitude of the advance made in this work. Because the many of the size measurements are indirect, it is not currently clear what magnitude of an effect SETD2 has on cell size. Moreover, it is inconclusive whether this is mediated by changes in cell cycle timing when SETD2 is removed. Because the cell size phenotype is not described in depth, it is not clear what the broader consequences of SETD2 on cell size regulation are. Moreover, the mechanistic insight into SETD2's function in this context is limited. Biochemically, because of the aforementioned issues with the SETD2 catalytic domain experiments, it is unclear how SETD2 is exerting this effect. More broadly, it is unclear whether SETD2 has an effect on cell size, if it is globally increasing translation rates, or if the effect is mediated through changes in transcription. As such, this manuscript provides an interesting and potentially important preliminary observation that requires further mechanistic investigation.

Comparison to existing published knowledge:

This manuscript uncovers a new connection between cell size, translation, and SETD2, which has no previously established role in these processes. Thus, this manuscript represents a new piece of information about SETD2's function.

Audience:

This manuscript is broadly interesting to members of the cell size regulation, histone, and epigenetics communities.

Your expertise:

My expertise falls into the cell size regulation, cell cycle, and proteostasis fields. As such, these are the aspects of this manuscript I am most qualified to discuss. Still, there are no pieces of this manuscript that I feel unqualified to review.

Author response to reviewers' comments

1. General Statements [optional]

This section is optional. Insert here any general statements you wish to make about the goal of the study or about the reviews.

SETD2 is a methyltransferase conserved from yeast to humans that is best known for its ability to methylate histone H3 on lysine 36 (H3K36) and thereby play various roles in chromatin biology. In recent years SETD2's function has been extended beyond chromatin regulation. In our manuscript, we find that SETD2 perturbation affects the size of human cell lines and that SETD2 depletion increases the global protein translation rate.

2. Description of the planned revisions

Insert here a point-by-point reply that explains what revisions, additional experimentations and analyses are planned to address the points raised by the referees.

Three major points are raised by the reviewers:

The first point regards the mechanism through which SETD2 controls cell size. We observe that SETD2 depleted cells have an increased protein content (per genome) and an increased protein synthesis rate (normalized to total protein content). In the discussion we suggest therefore that SETD2 might regulate translation, also given the reported role of the SETD2-opposing demethylase KDM4A in protein translation. The reviewers suggest a few experiments to explore other/additional

© 2022. Published by The Company of Biologists under the terms of the Creative Commons Attribution License (https://creativecommons.org/licenses/by/4.0/).

mechanisms of cell size regulation namely (1) through regulating the chromosome copy number (ploidy) of cells or (2) by regulating global RNA synthesis rates. These scenarios are interesting to explore, especially given that SETD2 methylates tubulin during mitosis (and thereby regulates genome stability; 10.1016/j.cell.2016.07.005) and that SETD2 is involved in transcription regulation. **To address these points we will first determine if inducible depletion of SETD2 results in changes in ploidy by determining chromosome copy numbers in metaphase spreads. Secondly, we will determine global RNA synthesis rate changes in SETD2 depleted cells by a pulse-labeling method.**

1. The reviewers point out that while we have directly measured cell size for WT vs SETD2 knock-down (imaging flow), for other perturbations we used protein content (normalized by genomic DNA) as a proxy for cell size. They reviewers recommend using precise definitions throughout the text and including direct cell size/volume measurements for other key perturbations. The reviewers also point out that for figures 3 and 4 - in which we perturb SETD2's function by overexpressing H3.3 mutants and the yeast and human SRI domains - more replicates are needed for statistical analysis. To simultaneously address both these issues **we will directly measure cell volume using a Casy counter of cells depleted for SETD2** (thereby complementing our findings in figure 1 and 2) **as well as cells overexpressing H3.3 mutants** (figure 3) **and SRI domains** (figure 4) **with adequate biological replicates** to determine if cell volume is affected under these conditions. We will also include cell volume measurements after SETD2 depletion for a cell line other than RPE1. Our pilot experiments with the Casy counter show that the increase in cell volume in SETD2 knock-down cells can be readily detected.

Related to this point, it is suggested that our findings could be strengthened by measuring protein content with an independent method such as amine-reactive dyes. **We will therefore set up a method to measure protein content per cell using fluorescent amine reactive dyes combined with flow-cytometry** as suggested by the reviewers. These dyes irreversibly react with cellular proteins and are typically used to distinguish live and dead cells from each other as these dyes are not permeable to living cells. For our purpose, we will first optimize a protocol to use these dyes for a quantitative measurement of the total protein content per cell. These experiments are aimed at further strengthening the findings in our manuscript.

2. We use a truncated SETD2 construct for increased SETD2 dosage and for rescue experiments. The reviewers make several suggestions to address uncertainties related to these experiments.

In figure 2 of our manuscript we overexpress a truncated SETD2 (tSETD2) to determine if we can rescue the effect on cell size we observe after microRNA-mediated depletion of endogenous SETD2 in RPE1 cells. We observe that cell size after SETD2 depletion is partially rescued in cells stably transduced with a miRNA-resistant tSETD2 construct, and that in the absence of SETD2 knockdown these cells have a smaller cell size compared to control cells. However, in these experiments we are unable to detect tSETD2 (which is tagged with a red fluorescent protein [RFP]) on western blot using RFP/GFP antibodies. We therefore cannot verify that tSETD2 is indeed expressed and how its levels compare to endogenous SETD2.

Our results indicate that SETD2 may affect cell size independent of its catalytic activity. However, we have not been able to test this idea directly. In our experience, overexpression of tSETD2 as well as catalytically inactive tSETD2-Q1669A by transduction is toxic to RPE1 cells and we believe that we therefore select for cells expressing low levels of tSETD2. SETD2 is normally also expressed at relatively low levels (an estimated 1000-9000 protein copies per cell in various mammalian cell lines 10.1074/mcp.M113.037309). We will therefore determine if we can detect RFP-tSETD2 by fluorescent microscopy, which will also inform us on the subcellular localization of tSETD2 as pointed out by one of the reviewers. Related to this, we will include images of the yeast SRI domain (which has an RFP tag) to show its subcellular localization.

As mentioned in our manuscript, we were unable to obtain a RPE1 cell line stably expressing catalytically inactive tSETD2-Q1669A. Reviewers point out that rescuing SETD2 knockdown by expressing a catalytically inactive tSETD2 is important to establish whether SETD2 controls cell size through its catalytic activity, and that we cannot conclude this based on the experiments in which we overexpress the KDM4A demethylase. **To address both the issue of tSETD2 detection**

© 2022. Published by The Company of Biologists under the terms of the Creative Commons Attribution License (https://creativecommons.org/licenses/by/4.0/).

and the toxicity of tSETD2(Q1669A), we will generate RPE1 cell lines stably expressing an inducible and HA-tagged version of WT tSETD2 and tSETD2(Q1669A). In combination with depletion of endogenous SETD2, we will determine the cell volume of inducible tSETD2(Q1669A) cells by Casy counter measurements. **In this way we aim to provide better evidence for the involvement of the catalytic activity of SETD2 in regulating cell size.**
The reviewers raise several minor points that we will address with minor modifications in the text.
Description of the revisions that have already been incorporated in the transferred manuscript
Please insert a point-by-point reply describing the revisions that were already carried out and included in the transferred manuscript. If no revisions have been carried out yet, please leave this section empty.

3. Description of analyses that authors prefer not to carry out
Please include a point-by-point response explaining why some of the requested data or additional analyses might not be necessary or cannot be provided within the scope of a revision. This can be due to time or resource limitations or in case of disagreement about the necessity of such additional data given the scope of the study. Please leave empty if not applicable.

Original submission

First decision letter

MS ID#: JOCES/2022/259856

MS TITLE: SETD2 negatively regulates cell size through its catalytic activity and SRI domain

AUTHORS: Thom M Molenaar, Eliza Mari Kwesi-Maliepaard, Joana Silva, Muddassir Malik, William Faller, and Fred van Leeuwen
ARTICLE TYPE: Research Article

We have now reached a decision on the above manuscript.

Thank you for providing the reviews that you received on your paper through Review Commons and a Review Plan. I have been through all of these and your manuscript and am happy for you to revise your paper as you lay out in your plan.

If you think that you can deal satisfactorily with the criticisms on revision, I would be pleased to see a revised manuscript. We would then return it to the reviewers.

We are aware that you may be experiencing disruption to the normal running of your lab that makes experimental revisions challenging. If it would be helpful, we encourage you to contact us to discuss your revision in greater detail. Please send us a point-by-point response indicating where you are able to address concerns raised (either experimentally or by changes to the text) and where you will not be able to do so within the normal timeframe of a revision. We will then provide further guidance. Please also note that we are happy to extend revision timeframes as necessary.

Please ensure that you clearly highlight all changes made in the revised manuscript. Please avoid using 'Tracked changes' in Word files as these are lost in PDF conversion.

I should be grateful if you would also provide a point-by-point response detailing how you have dealt with the points raised by the reviewers in the 'Response to Reviewers' box. Please attend to all of the reviewers' comments. If you do not agree with any of their criticisms or suggestions please explain clearly why this is so.

© 2022. Published by The Company of Biologists under the terms of the Creative Commons Attribution License (https://creativecommons.org/licenses/by/4.0/).

First revision

Author response to reviewers' comments

Thank you for reviewing our manuscript by Molenaar et al on the role of SETD2 on cell size control. The reviewers raised several very valuable suggestions that helped us to improve our manuscript.

Briefly, we have been able to make the core observation that SETD2 negatively cell size more robust by including direct measurements of cell volume and by repurposing amine-reactive dye-based flow- cytometry methods to measure cellular protein content. In addition we determined global nascent RNA synthesis rates and observed no evidence for a role of SETD2 in this potential aspect of cell size homeostasis. Finally, using an orthogonal approach (i.e. SETD2 protein degradation instead of knock- down) we now demonstrate that the role of SETD2 in cell size control is an evolutionarily conserved process in different cell types.

In the course of doing these experiments, we uncovered that H3.3K36A overexpression also led to a small increase in cell size (see Figure 4D), which calls into question our initial hypothesis that SETD2 regulates cell size independently of H3K36me3 (please see the response to comment 3 of reviewer #1 for details). We spent considerable time and effort probing this question further by expression of rescue constructs, as was also suggested by the reviewers (e.g. see response to reviewer 2). We made several attempts to express wild-type and catalytically inactive truncated SETD2 using constitutive and inducible expression settings. However, none of the approaches led to detectable expression of SETD2 protein, strongly suggesting that tSETD2 is difficult to transduce or that overexpression of wild-type or inactive tSETD2 is toxic to RPE1 cells, even when protein levels are suppressed. We have therefore decided to remove the experiments involving tSETD2 overexpression from the manuscript, as we can neither verify expression levels nor localization of tSETD2. In the light of these insights, we have adjusted the title of our manuscript to focus on the core findings of our study and not refer to the role of the catalytic activity of SETD2.

In the revised manuscript we changed the order of the authors and we added additional co-authors to reflect the substantial contributions made during the revisions. All authors agreed with the changes.

Please find below the point-by-point response to the comments.

Reviewer #1:

The manuscript by Thom M. Molenaar and colleagues reports a novel, unexpected role for the SETD2 methyltransferase in human cells. Through a series of elegant and well-controlled experiments, the authors show that SETD2 negatively regulates protein synthesis and cell size. Although their work did not identify which substrate is involved and how SETD2 inhibits translation, they bring evidence that this function is independent of H3K36 methylation but requires its catalytic activity. The paper is pleasant to read and well presented. However, I also think that the authors have to bring more compelling evidence to support their conclusions.

Major comments:

1. An important assumption in normalizing to genomic DNA levels to estimate total protein content is that genomic DNA equals cell number. However, this is true only if ploidy stays constant. Although using an inducible system minimizes the risk of ploidy changes, it is important to verify this within the time course of the Dox treatment (3 days) used here.
>> Changes in ploidy are indeed known to influence protein content and cell size. Methylation of alpha-tubulin by SETD2 during mitosis has also been reported to promote genome stability (Park et al., 2016 *Cell*), and protein content could therefore conceivably be altered in SETD2-depleted cells through changes in ploidy. We therefore analyzed chromosome spreads to look at changes in ploidy levels following inducible depletion of SETD2 (Figure 2A). As discussed on page 5, we did not find evidence for altered chromosome counts suggesting the changes in protein content and cell size are likely not due to changes in ploidy.

© 2022. Published by The Company of Biologists under the terms of the Creative Commons Attribution License (https://creativecommons.org/licenses/by/4.0/).

2. The effects seen upon gDNA normalization are modest (Figure 1 and 3), which can be expected for global changes in cell content / size. It is nevertheless critical to quantify the signal intensities from the Western blots used to build panels C and E, as well as their replicates, in order to give a more accurate view of the size and reproducibility of the effects. The authors should also provide evidence that the Western blotting were done in a semi-quantitative manner, for example by loading one sample at 2-3 dilutions. Also, Figure 3A would be strengthened with an analysis of b-actin levels, which showed stronger effects upon SETD2 knockdown (Figure 1).
>> Western blots are indeed semi-quantitative at best. Instead of focusing on quantifying more western blots, we have focused on independent quantitative methods to verify cell size increase following SETD2 perturbation. We have now included Casy counter measurements of cell volume with at least three biological replicates throughout the manuscript. We have confirmed that SETD2 perturbation using multiple strategies results in an increase in cell volume (Figure 1F, 1I, Sup Figures 2-3, Figure 4D, Figure 5D). We have also verified the increase in protein content in SETD2-depleted cells by repurposing a flow-cytometry method using amine-reactive dyes, adapting it to stain all proteins in permeabilized cells, which provides a more direct and quantitative method compared to western blotting to measure total protein content per cell (Figure 1G), lines 182-190. These new experiments support our initial observations with the western blots that short-term inducible SETD2 depletion leads to an increase in cell size/volume and a concordant increase in protein content.

3. One argument against a role of H3K36me3 in SETD2 function in protein content regulation is the comparison between the effects seen upon SETD2 knockdown (Figure 1A,D) and overexpression of the dominant-negative H3.3-K36M mutant (Figure 3B). It is difficult to directly compare data from different experiments / Western blots. Indeed, the differential effect on total protein content is weak: ~50% decrease for SETD2 knockdown compared to ~25% decrease for the H3.3 mutant. It is possible that there is the same difference for H3K36me3 levels. It is important to clarify this issue by running the SETD2 KD samples alongside the mutant H3.3K36 samples, on the same gel. Moreover, the experiment using the H3.3-K36A mutant is potentially more convincing, but there are no data showing its effect on total protein content and cell size. Both experiments are critical to substantiate the authors' conclusion that SETD2 controls cell size independently of H3K36me3.
>> We have now directly compared H3K36me3 levels in SETD2 knockdown cells versus H3.3K36A overexpressing cells (Supplementary Figure 4A). Cas13d-mediated knockdown of SETD2 lowers H3K36me3 substantially more than H3.3K36A overexpression, which is not surprising given that H3.3K36A has to compete with over a dozen unmodified histone H3 genes for total histone H3 levels. We have also measured cell volume in H3.3K36A overexpressing cells. We found a small increase in cell size (Figure 4D), which is something we did not pick up before using western blots for protein normalized lysates. The question therefore is if the minor increase in cell size in H3.3K36A overexpressing cells is due to (1) reduced levels of H3K36me3 (but not as reduced as in SETD2 knockdown cells, leading to a more modest phenotype), or (2) reduced activity of SETD2, i.e. towards both histone and non-histone substrates, as H3.3K36A has a minor trans inhibitory effect (Lu et al, 2016), and also note how H3.3K36A overexpression affects H3K36me3 levels on endogenous histone H3). Because we cannot resolve this question at this point, we have toned down our conclusion that SETD2 regulates cell size independently of H3K36me3. Our experiments highlight that it is difficult to functionally separate SETD2's substrates from each other, particularly histone substrates from non-histone substrates as it remains technically challenging to simultaneously mutagenize all histone H3 copies in human cells.

4. Figure 4B shows that overexpression of the yeast Set2 SRI domain increased total protein content, but there are no data on cell size, contrary to the conclusion on lane 318. Although these two readouts are obviously correlated, the paper would be stronger if cell size measurements were provided for key conditions, including the H3.3-K36A mutant and the yeast and human SRI decoy.
>> Thank you for the suggestion. As mentioned above, we have now measured cell volume in SETD2 knockdown cells, H3.3K36A overexpressing cells, and decoy SRI overexpressing cells.

5. A key experiment to support a role for SETD2 on protein synthesis would be to verify that SETD2 overexpression has an effect on cell size even if not localized in the nucleus. The authors tried this unsuccessfully with the SRY decoy, but employed a different inducible system (Shield), which may be more leaky than the Dox used in other experiments (Figure 1-3).

© 2022. Published by The Company of Biologists under the terms of the Creative Commons Attribution License (https://creativecommons.org/licenses/by/4.0/).

A properly inducible SETD2-NES or SRY-NES would bring compelling evidence supporting the central message of this paper.
>> We indeed reasoned as well that it would be very interesting to determine how inhibiting SETD2's function in different subcellular compartments would affect the cell size phenotype. Overexpression of a decoy SRI domain tagged with a NLS reduces H3K36me3 levels, suggesting that it inhibits SETD2 in the nucleus. Following this observation, we also wanted to inhibit SETD2's function in the cytoplasm (note that SETD2 has been reported to be both localized in the nucleus as well as the cytoplasm, Seervai et al., 2020). As mentioned in the results section (line 342-346), we have tried to express a NES-tagged (yeast) SRI decoy domain (without Shield inducible destabilizing domain [DD]), but we could not obtain cells stably expressing NES-SRI at high levels. We also established a cell line expressing DD-tagged NES-SRI to prevent toxicity by lowering protein levels (i.e. in absence of Shield- 1). However, treating these cells with Shield-1 did not result in the stabilization of DD-NES-SRI as it was still expressed at only a fraction of the levels of DD-NLS-SRI. This suggests that NES-SRI is either inherently unstable, or that there is a strong selection against cells expressing NES-SRI at high levels. We have removed the statement that SETD2 might target a component of the translation machinery.

Along this line, is there a properly normalized analysis (eg using spike-in controls) of nascent and total RNA content in ySet2/hSETD2 mutants, which the authors could use as further evidence that SETD2 control translation independently of an effect on chromatin?
>> Besides expression changes in specific genes that might contribute to the cell size phenotype, a global increase in transcription could conceivably also lead to an increase in cell size, which would indeed make spike-in controls essential in analyzing specific target genes. To look at global changes in transcription rates, we have adapted a protocol for transient transcriptome sequencing (TT-seq) using 4SU labeling of newly synthesized RNA to measure total RNA synthesis rates. We did not find changes in global RNA synthesis rates in SETD2-depleted cells using this assay. We now discuss this on page 5 line 216-223 and show the results in Figure 2B.

Minor comments:

Lane 123: "which differs five amino acids" is missing the preposition "by".
>> Thank you pointing this out; it is now corrected.

Lane 136: Although observing reduced levels of H3.3 upon SETD2 knockdown is indeed unexpected, I am not surprised by the global effect. Indeed, the human genome is not constituted of "only a small percentage" of "active gene bodies". The ENCODE studies showed that, in human cells for example, a large fraction of the genome is active (> 50%), consistent with the presence of large introns in most genes, which are transcribed and, therefore, decorated with H3K36me3.
>> Thank you for this remark. About 38% of the human genome consists of annotated coding and non-coding (e.g. lncRNAs) exons and introns – regions that can potentially be transcribed by RNAPII in a given cell type (i.e. not every gene is active in every cell type) (Rigau et al., 2019; https://doi.org/10.1371/journal.pgen.1007902). ENCODE studies have indeed suggested that an even larger part of the human genome is transcribed (Djebali et al. 2012). It is therefore somewhat surprising that mass-spec studies find that only 1-5% of histone H3 is marked by H3K36me3 in multiple cell lines (LeRoy et al., 2013). Factors that could explain this discrepancy are that not all RNAPII transcribed regions are decorated by H3K36me3 or that in individual cells not all nucleosomes in a H3K36me3-decorated region actually contain H3K36me3. We have now added a reference to acknowledge that a large part of the genome might be transcribed by RNAPII (page 4, lines 153-154).

It took me a long time to understand that the legends to Figure 1 and 2 are shifted: In the legend to Figure 1, (A) seems to describe what is labelled in panels A-C and (B) appears to describe panels D and E. The data described in (C) appear to be in Figure 2A etc...
>> We sincerely apologize for this error. We fixed the legends.

I suggest that the authors add a sentence acknowledging that the catalytically active truncated version of SETD2 (tSETD2) only partially restore H3K36me3 levels (Figure 1D) and might therefore not be fully active.

© 2022. Published by The Company of Biologists under the terms of the Creative Commons Attribution License (https://creativecommons.org/licenses/by/4.0/).

>> Because we could not verify that tSETD2 was indeed expressed in these cell lines, we have removed the experiment involving overexpression of tSETD2. Please see the response to Reviewer#2 for details.

The authors report "a minor decrease in the expression of genes involved in cell cycle progression such as E2F1/2, MCM5/6 and CDK6", which is not clearly visible on Figure 2D. E2F1/2 and MCM5 levels are stable, while MCM6 and CDK6 levels decrease, though upon targeting SETD2 with only one of the sgRNA, not the other. In contrast, the only visible and reproduce difference is a small increase of CDKN2A and TOP2A levels. Please modulate the interpretation of this experiment.
>> We have adjusted the text accordingly (top of page 6).

Lane 261: correct "is the they both act".
>> Fixed.

Can the authors explain why there is no band corresponding to HA-tagged H3K36me2 in the last lane of Figure 3B (upon overexpression of H3.3-G34R-HA)?
>> The mutant H3.3-G34R prevents not only tri-methylation by SETD2, but also di-methylation by the methyltransferase NSD2. Therefore, the tagged (upshifted) mutant protein is not di-methylated. We explain this now more clearly in the text (top of page 7).

The data shown in Figure 4C show that the SRY-R2510H mutation reduces the effect observed with the WT SRY domain, but does not "abolishes" (lane 348) its function. Please rephrase.
>> To be more accurate, we changed abolishes to reduces (line 360).

Lane 380: for clarity, I suggest to state more explicitly that SETD2 inhibits protein synthesis and cell growth (ie. size).
>> Thank you for your suggestion. We have changed ‘regulation’ to ‘inhibition’ (line 379).

All statistical analyses were performed using a Student t-test, which is not appropriate to compare more than two means. Rather, an analysis of variance (ANOVA) followed by post-hoc comparisons, which is available in Prism, is designed to compare more than two samples. In addition, the data shown in Figure 3A, 3B and 4C have only two data points and should be re> produced more than once.
>> Where appropriate, we have now used ANOVA for statistical analysis of experiments using three biological replicates.

Reviewer #2

Summary
This manuscript describes a novel role for the histone methyltransferase SETD2 in regulation of cell size. SETD2 is a conserved methytransferase for histone H3 lysine 36 and is frequently mutated in cancer. It has mostly been studied as the catalyst for methylation of histone H3, although it has other targets in mammalian cells. The authors used conditionally expressed miRNA to knock down SETD2 in RPE1 cells. This led to the expected decrease in H3K36me3 levels, but also decreased total H3 and H4 levels with respect to abundant cytoplasmic protein markers such as actin and tubulin. The authors further showed a decrease in the ratio of DNA to unit protein in SETD2 knockdown cells. Overexpression of a catalytically active SETD2 had the opposite effect. The authors determined that SETD2 knockdown led to increased cell size (measured by flow cytometry) and protein synthesis rates. Overexpression decreased cell size. This effect on cell size was apparently independent of an effect on cell cycle progression. To determine the contribution of H3K36me to these effects, the authors overexpressed demethylase enzymes. A yeast enzyme that specifically acts on H3K36me3 did not affect DNA/protein ratios, whereas KDM4A, which acts on histone and non-histone SETD2 substrates, had a similar effect as SETD2 knockdown. Overexpression of H3.3 K36 mutants failed to phenocopy SETD2 knockdown. In contrast, overexpression of the SRI domain, which directs SETD2 activity to substrates through protein-protein interactions, did affect DNA/protein ratios, presumably by competing with endogenous SETD2. The authors conclude that SETD2 regulates cell size through methylation of a substrate other than H3K36.

© 2022. Published by The Company of Biologists under the terms of the Creative Commons Attribution License (https://creativecommons.org/licenses/by/4.0/).

Major comments
The manuscript is clear and concise and the data generally support the conclusions. The only "major" comment I have is to provide more clarity surrounding experiments in which SETD2 fragments are overexpressed. Here it is important to know both the levels and subcellular distributions of the overexpressed constructs and how they compare to endogenous SETD2 as there are implications for which SETD2 targets are being affected. For example, the SRI domain overexpression experiments used SRI domain fused to an SV40 NLS-does this mean the decreased DNA/protein ratio is connected to a nuclear target? The Discussion favors other possibilities eg ribosomal proteins. Did other expression constructs also include an NLS? Subcellular localization should be verified for these experiments as well.
>> Thank you for your review of our manuscript. In trying to address this important point, a major challenge we had with the experiments involving stable overexpression of tSETD2 was that we could not verify that (RFP-tagged) tSETD2 was actually expressed. Although cells were resistant to the selection marker on the tSETD2 vector, we could not verify by fluorescent microscopy or western blot that cells actually expressed tSETD2 (using either RFP/GFP antibodies or a SETD2 antibody).
Because H3K36me3 levels also did not change detectably (especially when looking at DNA normalized western blots), we have decided to remove the experiments involving tSETD2 overexpression. We have made several attempts to make new cell lines by transducing cells with a new tSETD2 construct. In the old tSETD2 construct, SETD2 lacked the first 500 amino acids (part of the large unstructured N-terminal domain, which negatively regulates SETD2 stability (Bhattacharya et al. 2021)). For the revision experiments, we removed the N-terminal domain completely (removing the first 1400 amino acids of SETD2). This version of SETD2 (tSETD2[1400-2564]) has been reported to be more stable when ectopically expressed and is still functional (Bhattacharya et al.
2021). We tagged this new tSETD2[1400-2564] with a destabilizing domain (DD) for Shield-1 inducible expression and an HA-tag for detection. However, similar as with the RFP-tSETD2 construct, we only obtained a few colonies following transduction and selection, with both a wt and catalytically inactive (Q1669A) SETD2, and we could not detect tSETD2 expression by western blot in the colonies we obtained after transduction. This suggests that either tSETD2 is difficult to transduce or that tSETD2 overexpression is toxic to RPE1 cells, even when protein levels are suppressed using the DD, or when tSETD2 is catalytically inactive. We have therefore decided to remove the experiments involving tSETD2 overexpression from the manuscript, as we can neither verify expression levels nor localization of tSETD2.

Regarding the SRI overexpression experiments, our finding that ectopic SRI fused to an NLS increases cell size indeed suggests that this phenotype is related to a nuclear target. We have now included images from live-cell microscopy to show the nuclear localization of RFP-NLS-SRI(R2510H) in RPE1 cells (Figure 5B). Other expression constructs such as KDM4A and Rph1 did not include extra NLSs. It may be good to note that ribosomes are assembled in the nucleus (in the nucleolus) and thus could still be potentially targeted by SETD2. However, given that we do not have any direct evidence for SETD2-mediated methylation of a ribosomal component, we have removed the paragraph where we discuss a potential link between SETD2 and the translation machinery from the Discussion section.

Minor comments
-Figure 1 legend is wrong. The description does not match what is in the figure.
>> We sincerely apologize for this error. We fixed the legends.

-no method provided for determination of protein concentration
>> We now mention the method used to determine protein concentrations (bottom of page 12).

-Figure 2 legend is out of order
>> We sincerely apologize for this error. We fixed the legends.

-Figures 3A and B are based on two repeats-are any of these differences significant?
>> We have indeed only two repeats for the KDM4A and H3.3K36M overexpression experiments. Our aim with these experiments was to functionally separate histone vs non-histone methylation by SETD2. However, KDM4A might also demethylate non-histone targets and similarly H3.3K36M might also affect non-histone substrate methylation as it inhibits SETD2 in trans. We therefore have

© 2022. Published by The Company of Biologists under the terms of the Creative Commons Attribution License (https://creativecommons.org/licenses/by/4.0/).

directed more focus towards the H3.3K36A overexpression experiments, for which we now have included three replicate measurements of cell volume (Figure 4D).

-there is nothing in 3C (or anywhere else) showing the effect of H3.3K36A overexpression on DNA/protein ratio
>> We now directly measure the cell volume of H3.3K36A overexpressing cells (Figure 4D).

-more needs to be said about the experiment in Figure 4C. The data as shown are difficult to understand. Why is there no effect of the shield 1 compound on the ability of DD-hSRI expression to phenocopy SETD2 depletion, despite the fact that in the absence of shield 1 expression is barely detected?
>> The destabilizing domain (DD) does not completely block protein expression in absence of Shield-**3.** We have now repeated this experiment using RFP- instead of DD-tagged hSRI constructs (Figure 5). This circumvents both the need to induce protein stabilization using Shield-1 and also allows us to look at live-cell localization of the ectopic SRI domains. We find the same effect as with the DD- tagged hSRI constructs, namely that hSRI overexpression increases cell volume, whereas the R1510H mutant does not. We also confirm that RFP-NLS-hSRI is localized in the nucleus (Figure 5B).

-subtle effect of Shield1 compound in 4C for the SRI point mutant is not convincing based on two repeats-need to be able to show the stats
>> We now directly measure the size of SRI overexpressing cells, using three biological replicates and statistical analysis (Figure 5D).

Reviewer #3:

Summary:
In this manuscript, Molenaar, et al. investigate the role for SETD2 (a lysine methyltransferase) in cell size regulation. They show that SETD2 depletion changes the ratio of genomic DNA to total protein and ascribe this to changes in cell size. They then set out to uncover whether the catalytic activity of SETD2 and the SRI interaction domain are required for mediating this phenotype. The authors proceed to conclude that the role of SETD2 in regulating cell size requires its catalytic activity but is independent of H3K36 methylation.

While the separation of function experiments that were conducted to probe the importance of the SRI domain were clever and well done, there are several issues with the way the effect of SETD2 on cell size is quantified, making it unclear how much of a role SETD2 truly has on regulating cell size. Moreover, the authors do not directly show that SETD2's catalytic activity is required for this observation, making it difficult to draw the conclusion that SETD2 regulates cell size through its catalytic activity. Together, these issues bring into question the magnitude of the effect of SETD2 on cell size and the mechanistic basis for how this mode of regulation occurs.

<u>Major Comments:</u>
1) Size measurements should be made directly and not by comparing protein ratios. The effect of SETD2 on cell size is an extremely important point in this manuscript, however there is only one figure (Figure 2A) that shows any measurement of this. Moreover, the technique used for this (imaging flow cytometry) is not explained either in the text or in the methods and deviates from those that are commonly found in publications about cell size (e.g., Coulter Counter-based measurements, QPI, or measurement of protein content using amine-reactive dyes). Because cell volume is relevant here, it would be important to include measurements of cell volume (instead of just area). Based on the provided measurements, it is not intuitive what the indicated difference in size means relative to the control cells (e.g., how many times bigger are the SETD2 knockdown cells relative to the control?). It would be important for the authors to show this phenotype using another method and to include these measurements in all experiments that claim changes in cell size.

>> Thank you for the suggestion to include methods directly measure cell size. We have now included new Casy counter-based measurements of cell volume of conditions in which we perturbed SETD2 using different strategies including SETD2 knockdown (Figure 1F, 1I and

© 2022. Published by The Company of Biologists under the terms of the Creative Commons Attribution License (https://creativecommons.org/licenses/by/4.0/).

Supplementary Figure 1B, 2), H3.3K36A overexpression (Figure 4D), and SRI decoy overexpression (Figure 5D). We have also performed new experiments using an amine-reactive dye to more quantitatively and directly measure protein content in SETD2-depleted cells (Figure 1G). Both sets of experiments confirm our initial findings that SETD2 depletion increases cell size and protein content. Using these new, more quantitative measurements, we estimate that inducible loss of SETD2 increases cell size and protein content by ~30-40%, which we now explicitly state in the manuscript (bottom of page 4).

2) Total protein quantifications: The authors show that the effect on cell size correlates with an increase in protein content and translation rates. However, cell size and protein abundance are not synonymous measurements, and one should not be used as a proxy for the other (as is the case in Figure 4B or lines 273-276, for example). Moreover, the measurement of total cellular protein by normalizing to genomic DNA could be more convincingly made by Coomassie staining rather than by looking at the loss of histone proteins when gel loading is normalized to protein content vs. maintenance of histones when normalized to genomic DNA. As mentioned in the previous comment, amine-reactive dyes may also be useful for this.
>> We agree that cell size can change without any effect on protein content, e.g. through osmotic effects. As mentioned above, we have developed a flow-cytometry based amine-reactive dye staining protocol (using the live-dead Zombie dyes) to measure the total protein content of cells that were fixed and permeabilized. The results from this experiment and the genomic DNA : total protein ratio both point towards an increased protein content following inducible depletion of SETD2.

3) The authors indicate that SETD2 controls cell size through its catalytic activity, however this is not shown directly. They instead show opposing effects when KDM4A (a demethylase with activity that counteracts SETD2) is expressed. While these results are promising for their proposed mechanism, they do not directly show that SETD2's catalytic activity is the driver of the observed phenotype. Moreover, the effect of KDM4A on protein synthesis is already established (Van Rechem et al. 2015), therefore this observation does not add any new insight. As such, it is misleading to definitively assign importance to SETD2's catalytic domain based on these data. While the authors indicate they had trouble generating a stably overexpressing catalytically inactive tSETD2, they may consider generating a doxycycline-inducible one instead.
>> Following your suggestion, we have removed our initial interpretation that the KDM4A overexpression experiment indicates that SETD2 regulates protein content through its catalytic activity from the manuscript. KDM4A could indeed also control cell size through mechanisms independent of SETD2, e.g. through effects on heterochromatin via H3K9 methylation. The best way to show the potential involvement of SETD2’s catalytic activity would indeed be a rescue experiment with catalytic dead tSETD2. We have experienced that Dox-inducible expression of large cDNAs typically results in a very heterogenous expression pattern in a population of cells (i.e. the system is very leak-proof, but some cells don’t express the cDNA at all following Dox treatment). We reasoned that DD-tagging of a new, more truncated version of SETD2 (lacking the first 1400 amino acids, which is more stable than tSETD2 lacking the first 500 amino acids; (Bhattacharya et al. 2021)) would give us an inducible version of tSETD2 that would be more stable once induced. However, as explained in more detail at the response to Reviewer #2 above, we were still not able to generate stable cell lines using these new constructs, either because of the leakiness of the DD and toxicity of even low levels of (catalytically inactive) tSETD2, or because of inefficient transduction of the lentivirus. We therefore do not have definitive evidence for the involvement of SETD2’s catalytic activity in regulating cell size. We have removed this claim from the results section and abstract.

4) The authors focus on total cellular protein abundance changes when SETD2 is knocked down but do not address the possibility that SETD2 is globally changing transcript abundance, which is in turn translating to a difference in protein amount. This seems especially important given SETD2's known relationship with RNAPII. Mechanistically, it would be important to show that SETD2 does not affect global transcript levels before focusing on total protein content.
>> Following your suggesting we have measured global RNA synthesis rates using 4SU labeling of nascent RNA in SETD2-depleted RPE1 cells (Figure 2B). We do not find significant differences in total RNA synthesis rates following SETD2 depletion (normalized for total RNA, similar as to how we normalize protein synthesis rates to protein content in Figure 2C). We acknowledge that

© 2022. Published by The Company of Biologists under the terms of the Creative Commons Attribution License (https://creativecommons.org/licenses/by/4.0/).

effects on RNAPII could be obscured because the bulk of new RNA might be RNAPI derived ribosomal RNA.

5) The authors show that bulk translation is increased in SETD2 knockdown cells. It would be important to establish whether this reflects an overall increase in protein synthesis due to increased size (with no change in the translation rate of specific transcripts) or if this increase is a specific effect that affects particular proteins.
>> It would indeed be interesting to determine if the translation rate of specific mRNAs is preferentially affected by SETD2, e.g. using ribo-seq. However, given that there is not yet a direct link between SETD2 and protein synthesis (i.e. there is a link between KDM4A and protein synthesis (Van Rechem et al., 2015) and an indirect link between SETD2 and translation efficiency (Hapke et al., 2020 preprint), we feel that this currently falls beyond the scope of our manuscript and we have not performed ribo-seq in SETD2 depleted cells. We would like to emphasize that the 35S-methionine incorporation measurements in Figure 2C are normalized to total cellular protein content, which suggests there is an increase in protein synthesis rate following SETD2 depletion that is not simply a consequence of the increased cell size/protein content itself.

Minor Comments:
1) The figure legends are off in a few places in this manuscript (e.g., Figure 1B and C do not match the legend, and there is no legend for Figure 1D-E; Figure 2 starts at 2B but is labeled as 2A)
>> We sincerely apologize for this error. We fixed the legends.

2) In the text, the authors indicate that there is a small decrease in the expression of E2F1/2, MCM5/6, and CDK6 when SETD2 is knocked down, but this is not what the bar plot in Figure 2D shows. Moreover, there seems to be a fairly robust increase in CDKN2A and TOP2A when SETD2 is knocked down, but this is not addressed in the text. Particularly because CDKN2A encodes CDK inhibitors that cause G1 cell cycle arrest, this seems like an important consideration given that the authors are trying to show a negligible effect on G1 duration. Based on these discrepancies, the cell cycle analysis that was conducted in Figure 2 seems to be inconclusive.
>> We have repeated the cell cycle distribution analysis in RPE1 cells in which SETD2 was depleted using RfxCas13d (Figure 2D), as well as in mESCs that have a Setd2-dTAG knock-in (Figure 2F). In these cell lines, we did not find significant differences in cell cycle distribution following SETD2 depletion. The increase in CDKN2A expression therefore does not appear to correlate with a consistent accumulation of cells in G1. Still, we now more clearly discuss the increase in CDKN2A expression in both the results (top of page 6) and discussion section (pages 8-9). Increased CDKN2A expression is an interesting lead for finding out how SETD2 loss increases cell size, given that p16 (one of the proteins encoded by CDKN2A) is a CDK4/6 inhibitor. We now discuss how finding out if there is a link between CDKN2A expression and the increased cell size following SETD2 depletion will require more careful measurements of G1 duration (page 9).

3) The method for how the cell cycle profiles were obtained in Figure 2C is not described in the text or in the methods. The raw FACS data for this (as well as the imaging flow cytometry experiment) should be included in the supplement.
>> We have included raw data for the flow-cytometry experiment to measure cell cycle distribution in the Supplementary Figures.

4) The initial description of SETD2's effect on the DNA: protein ratio is a bit confusing (lines 141-149). By stating that they ..."found that SETD2 depleted lysates had lower DNA levels", it seems to imply that SETD2 KD cells are losing DNA (which is not the case). The description of these experiments could be clarified.
>> Thank you for pointing this out, we now state that SETD2-depleted lysates had lower DNA levels *per protein content* (line 161).

5) The decrease in histones H3 and H4 described in the text and shown in Supplemental Figure 1C is not clearly visible by eye; thus, authors should include quantifications for multiple replicates if they wish to make the claim that this decrease is observable in multiple cell lines.
>> We have removed the figure in which we knockdown SETD2 in TIG3 and BJ cells, as the knockdown efficiency was not very high. To independently confirm our results in another cell type, we instead focus on mouse embryonic stem cells with Setd2-dTAG knock-in to ablate SETD2

© 2022. Published by The Company of Biologists under the terms of the Creative Commons Attribution License (https://creativecommons.org/licenses/by/4.0/).

by protein degradation instead of knock-down of mRNA expression. We observe a consistent effect of SETD2 depletion on cell size (Figure 1I) (lines 193-201), in two independent knock-in clones, suggesting that cell size increase following loss of SETD2 is not unique to human RPE1 cells.

6) There are several bar plots (e.g., Figure 1B, 2B, 2C) where the error bars are not identified (e.g., SEM or SD) and it is not indicated how many replicates were performed for these experiments.
>> Thank you for pointing this out. We have indicated at each figure what the error bars mean and how many replicates were performed.

7) In line 293, the authors state that "H3.3K36A did not affect cell size," however I cannot find data in the figures that support this claim.
>> We have now directly measured cell size of H3.3K36A overexpressing cells using a Casy Counter (Figure 4D) (lines 320-328).

8) The serum starvation experiment described in lines 287-290 is informative but as it is a negative result, putting it in the supplement may help streamline the text a bit.
>> We agree that this in essence a negative result. However, we prefer to keep it in the main text as we would otherwise have to make an extra supplementary figure without much improving the flow of the text.

9) For western blots that are normalized by genomic DNA, the authors should include the GAPDH qPCR results in the supplement.
>> qPCR was used to measure genomic DNA content of protein-normalized whole cell extract lysates. By determining the number of copies of a given gene in the lysate, we obtain a value for the number of genomes and hence a proxy for the amount of genomic DNA in the lysate. In this type of analysis, the relative raw values are used without any normalization for reference genes. This approach is in essence similar to how “input” samples are prepared and quantified for chromatin immunoprecipitation experiments when IP vs Input is measured. The qPCR data presented below the western blots is therefore represents the “raw” gDNA content of the lysates, and the only post- hoc normalization is to set a control (miRNA SETD2#1 -Dox) to 1.

Second decision letter

MS ID#: JOCES/2022/259856

MS TITLE: The histone methyltransferase SETD2 negatively regulates cell size

AUTHORS: Thom M Molenaar, Muddassir Malik, Joana Silva, Ning Qing Liu, Judith H.I. Haarhuis, Christina Ambrosi, Eliza Mari Kwesi-Maliepaard, Tibor van Welsem, Tuncay Baubec, William Faller, and Fred van Leeuwen
ARTICLE TYPE: Research Article

We have now reached a decision on the above manuscript.

To see the reviewers' reports and a copy of this decision letter, please go to: https://submit-jcs.biologists.org and click on the 'Manuscripts with Decisions' queue in the Author Area. (Corresponding author only has access to reviews.)

As you will see, the reviewers gave favourable reports but reviewers 2 and 3 raised some small points that I would like you to address. I hope that you will be able to carry these out because I would like to be able to accept your paper, depending on further comments from reviewers.

Please ensure that you clearly highlight all changes made in the revised manuscript. Please avoid using 'Tracked changes' in Word files as these are lost in PDF conversion.

© 2022. Published by The Company of Biologists under the terms of the Creative Commons Attribution License (https://creativecommons.org/licenses/by/4.0/).

I should be grateful if you would also provide a point-by-point response detailing how you have dealt with the points raised by the reviewers in the 'Response to Reviewers' box. Please attend to all of the reviewers' comments. If you do not agree with any of their criticisms or suggestions please explain clearly why this is so.

Reviewer 1

Advance summary and potential significance to field

This manuscript describes a novel role for the histone methyltransferase SETD2 in regulation of cell size. SETD2 is a conserved methytransferase for histone H3 lysine 36 and is frequently mutated in cancer. It has mostly been studied as the catalyst for methylation of histone H3, although it has other targets in mammalian cells. The authors used conditionally expressed miRNA to knock down SETD2 in RPE1 cells. This led to the expected decrease in H3K36me3 levels, but also decreased total H3 and H4 levels with respect to abundant cytoplasmic protein markers such as actin and tubulin. The authors further showed a decrease in the ratio of DNA to unit protein in SETD2 knockdown cells. The authors determined that SETD2 knockdown led to increased cell volume and protein synthesis rates, but not RNA synthesis rates. This effect on cell size was apparently independent of an effect on cell cycle progression or ploidy. To determine the contribution of H3K36me to these effects, the authors overexpressed demethylase enzymes. A yeast enzyme that specifically acts on H3K36me3 did not affect DNA/protein ratios, whereas KDM4A, which acts on histone and non-histone SETD2 substrates, had a similar effect as SETD2 knockdown. Overexpression of H3.3 K36 mutants produced a cell size increase that was less pronounced than that observed upon SETD2 knockdown. Overexpression of the SRI domain, which directs SETD2 activity to substrates through protein-protein interactions, strongly affected DNA/protein ratios and cell volume, presumably by competing with endogenous SETD2. The authors conclude that SETD2-dependent regulation of cell size may involve methylation of H3K36 in addition to non-histone substrates.
This manuscript is potentially significant because it clearly describes a novel cell size effect associated with SETD2 depletion, convincingly establishes protein synthesis as the likely driver of the effect, and makes some inroads into establishing the mechanism.

Comments for the author

The authors have responded to all of the previous reviews and have provided important confirmatory data supporting the effects on cell size. Suitable for publication as is.

Reviewer 2

Advance summary and potential significance to field

In this manuscript Molenaar et al. provide evidence that depleting the methyltransferase SETD2 from human cells leads to an increase in growth rate and cell size. These conclusions are well supported by the data. The mechanism by which SETD2 leads to an increase in cell size is however not clear. There is conflicting evidence on whether the increase in cell size is caused through the role of SETD2 on methylating Histone H3 K36 or through another, yet unidentified, SETD2 target: Overexpression of H3 K36A leads to a slight increase in cell size, suggesting that SETD2 regulates size through histone methylation. On the other hand, overexpression of a mutated SRI domain is sufficient to block H3K36 methylation (Figure 5C) but does not lead to an increase in cell size, suggesting a histone independent role.

Comments for the author

Despite these unresolved issues, the current manuscript was greatly improved compared to the initial pre-print submission and the authors have addressed most of my previous concerns. In particular, the conclusions on cell size are now based on direct size measurements. They have also toned-down or removed conclusions which were not fully supported by the data. In my eyes there are still two points that are unclear and need to be addressed:

© 2022. Published by The Company of Biologists under the terms of the Creative Commons Attribution License (https://creativecommons.org/licenses/by/4.0/).

In line 294 the authors state ”Taken together, these results so far suggest that SETD2 regulates cell size through its methylation activity but argue against direct involvement of its activity toward H3K36.” I do not see how the data presented in Figure 4A supports this statement. Overexpression of yeast Rph1 only partially reduces H3K36 and H3K9 methylation and causes a small but not significant reduction in cell size. Given the incomplete removal of H3 methylation and the partial size phenotype I don’t think one can conclude that the altered methylation pattern does not affect cell size.
The second point that is not clear to me is the role of the SRI domain. The authors state in line 361/362 “Surprisingly, SRI-R2510H overexpression did not strongly affect 362 H3K36me3 levels and also did not affect cell volume (Figure 5C, D).” However, in Figure 5C, H3K36me3 levels are clearly reduced in the SRI-R2510H expressing cells compared to the control. The authors should not make this claim as it is not supported by the data shown or present additional evidence that supports their conclusion. IF SRI-R2510H indeed suppresses H3K36 methylation, this would in my eyes indicate that the size phenotype is independent of histone methylation as cell size is not affected by SRI-R2510H.

Reviewer 3

Advance summary and potential significance to field

The manuscript by Thom M. Molenaar and colleagues reports a novel, unexpected role for the SETD2 methyltransferase in human cells. Through a series of elegant and well-controlled experiments, the authors show that SETD2 negatively regulates protein synthesis and cell size. These findings are unexpected and indicate that SETD2 has functions and substrates beyond its well-characterized roles during transcription elongation by RNA polymerase II. Although their work did not identify which substrate is involved and how SETD2 inhibits protein synthesis, this a very interesting manuscript that brings a new and unexpected contribution to our understanding of SETD2 activities and will be of great interest for the chromatin and cell size control fields. The paper is pleasant to read and well presented.

Comments for the author

Here I went through the revised manuscript and appreciate the efforts made to address the concerns raised during the evaluation of the preprint at Review Commons. The authors were able to strengthen the manuscript with additional experiments that, overall, substantiate their conclusions. I am particularly pleased by the work done to provide orthogonal, quantitative measurements of cell volume and total protein content. I also appreciate the caution taken in interpreting the H3K36 mutant phenotypes. I thus recommend publication and would like to congratulate all authors for the work done and this interesting story.
One small suggestion, for clarity, is to reference Figure 4C and Supplementary Figure 4A when describing "…that ectopic expression by the strong EEF1A1 promoter led to high incorporation levels of ectopic HA tagged H3.3 (H3.3-HA)." (Lane 316-137).

Second revision

Author response to reviewers' comments

Reviewer 2 Comments for the Author:
Despite these unresolved issues, the current manuscript was greatly improved compared to the initial pre-print submission and the authors have addressed most of my previous concerns. In particular, the conclusions on cell size are now based on direct size measurements. They have also toned-down or removed conclusions which were not fully supported by the data. In my eyes there are still two points that are unclear and need to be addressed:

In line 294 the authors state ”Taken together, these results so far suggest that SETD2 regulates cell size through its methylation activity but argue against direct involvement of its

© 2022. Published by The Company of Biologists under the terms of the Creative Commons Attribution License (https://creativecommons.org/licenses/by/4.0/).

activity toward H3K36." I do not see how the data presented in Figure 4A supports this statement. Overexpression of yeast Rph1 only partially reduces H3K36 and H3K9 methylation and causes a small but not significant reduction in cell size. Given the incomplete removal of H3 methylation and the partial size phenotype I don't think one can conclude that the altered methylation pattern does not affect cell size.
>> We agree with the reviewer that the data are not conclusive on this issue. Therefore, we now rephrased the text to: "Taken together, these results so far suggest that SETD2 regulates cell size through its methylation activity. However, given the incomplete removal of H3K36me3 in Rph1 overexpressing cells, we cannot definitively determine whether or not H3K36 methylation itself regulates cell size."

The second point that is not clear to me is the role of the SRI domain. The authors state in line 361/362 "Surprisingly, SRI-R2510H overexpression did not strongly affect H3K36me3 levels and also did not affect cell volume (Figure 5C, D)." However, in Figure 5C, H3K36me3 levels are clearly reduced in the SRI-R2510H expressing cells compared to the control. The authors should not make this claim as it is not supported by the data shown or present additional evidence that supports their conclusion. IF SRI-R2510H indeed suppresses H3K36 methylation, this would in my eyes indicate that the size phenotype is independent of histone methylation as cell size is not affected by SRI-R2510H.
>> We agree with the reviewer that SRI-R2510H overexpression does have an effect on H3K36me3 levels. We now mention this more explicitly in the text. We also now state that this could indeed be seen as evidence for involvement of H3K36me3 in regulating cell size. However, H3K36me3 is still not completely gone, so we remain careful with our conclusions and we explain the limitations of using the SRI-R2510H protein as a separation-of- function mutant.

Reviewer 3 Comments for the Author:
Here I went through the revised manuscript and appreciate the efforts made to address the concerns raised during the evaluation of the preprint at Review Commons. The authors were able to strengthen the manuscript with additional experiments that, overall, substantiate their conclusions. I am particularly pleased by the work done to provide orthogonal, quantitative measurements of cell volume and total protein content. I also appreciate the caution taken in interpreting the H3K36 mutant phenotypes. I thus recommend publication and would like to congratulate all authors for the work done and this interesting story.

One small suggestion, for clarity, is to reference Figure 4C and Supplementary Figure 4A when describing "...that ectopic expression by the strong EEF1A1 promoter led to high incorporation levels of ectopic HA tagged H3.3 (H3.3-HA)." (Lane 316-137).
>> Thank you for the suggestion. We now refer to Figure 4C and Supplementary Figure 4A as suggested by the reviewer.

Third decision letter

MS ID#: JOCES/2022/259856

MS TITLE: The histone methyltransferase SETD2 negatively regulates cell size

AUTHORS: Thom M Molenaar, Muddassir Malik, Joana Silva, Ning Qing Liu, Judith H.I. Haarhuis, Christina Ambrosi, Eliza Mari Kwesi-Maliepaard, Tibor van Welsem, Tuncay Baubec, William Faller, and Fred van Leeuwen
ARTICLE TYPE: Research Article

Thank you for sending your manuscript to Journal of Cell Science through Review Commons.

I am happy to tell you that your manuscript has been accepted for publication in Journal of Cell Science, pending standard ethics checks.

© 2022. Published by The Company of Biologists under the terms of the Creative Commons Attribution License (https://creativecommons.org/licenses/by/4.0/).
